# Foundation Model‐Enabled Multimodal Deep Learning for Prognostic Prediction in Colorectal Cancer with Incomplete Modalities: A Multi‐Institutional Retrospective Study

**DOI:** 10.1002/advs.202510931

**Published:** 2026-01-20

**Authors:** Linhao Qu, Chengsheng Zhang, Yingyong Hou, Feng Tang, Weiqi Sheng, Dan Huang, Zhijian Song

**Affiliations:** ^1^ Digital Medical Research Center, School of Basic Medical Sciences Fudan University Shanghai China; ^2^ Shanghai Key Laboratory of Medical Imaging Computing and Computer Assisted Intervention Shanghai China; ^3^ Department of Pathology Zhongshan Hospital Fudan University Shanghai China; ^4^ Department of Pathology, Huashan Hospital Fudan University Shanghai China; ^5^ Department of Pathology Fudan University Shanghai Cancer Center Shanghai China; ^6^ Department of Oncology, Shanghai Medical College Fudan University Shanghai China; ^7^ Department of Rheumatology Zhongshan Hospital Fudan University Shanghai China

**Keywords:** artificial intelligence, colorectal cancer, foundation model, multimodal deep learning, prognosis prediction

## Abstract

Accurate prognostic prediction for colorectal cancer is essential for optimizing personalized treatment strategies and improving patient outcomes. Current unimodal approaches encounter significant limitations in effectively leveraging multimodal data and confront challenges with the issue of missing modalities. A novel multimodal deep learning framework named FLARE, which integrates pathological images, radiological imaging, and clinical text reports, is introduced to provide accurate risk assessments for colorectal cancer survival and progression. FLARE employs foundation models to achieve efficient feature extraction, utilizes an attention‐based multi‐branch framework to enhance synergy and distinctiveness across modalities, and incorporates a diversity‐promoting loss function. To address the issue of incomplete data, FLARE integrates modality and missing‐aware prompts, pseudo embeddings, and a modality‐level augmentation strategy, thereby effectively mitigating potential performance degradation. The performance of FLARE is retrospectively assessed using a dataset of 1679 colorectal cancer patients from four independent clinical centers. Its superior prognostic capability is demonstrated through Kaplan‐Meier analysis and the concordance index. FLARE effectively stratified patients into high‐ and low‐risk groups. It achieved the highest concordance index across all validation cohorts, significantly outperforming traditional clinical models and existing multimodal methods, thereby highlighting its robust generalizability. Interpretability was enhanced by the comprehensive analyses of clinical factors, immune infiltration patterns, and gene pathways, as well as visualizations of feature importance across multiple modalities. In summary, FLARE establishes a comprehensive and robust framework for multimodal deep learning in medical prognostics, providing an advanced Artificial intelligence, Multimodal Deep Learning, Prognosis prediction, colorectal cancer, foundation modeltool for precision cancer prognosis and intelligent diagnosis.

## Graphical abstract

FLARE, a multimodal AI framework, combines pathology slides, radiology scans, and clinical reports to predict colorectal cancer outcomes, even when some tests are missing. Evaluated retrospectively in 1679 patients from four medical centers, it consistently achieved the best prognostic accuracy and clearly separated high‐ and low‐risk groups. Analyses of clinical factors and biological pathways add interpretability.

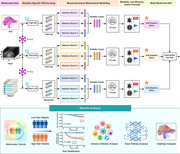

## 1 Introduction

Accurate prognostic prediction for colorectal cancer (CRC), encompassing both survival rates and disease progression, is of paramount importance for guiding treatment decisions and follow‐up strategies. Nevertheless, it still remains a significant challenge [1, 2, 3, 4, 5]. Current methods, such as the AJCC and TNM staging, predominantly depend on pathological staging approaches that are inherently subjective and quite limited, frequently neglecting individual patient‐specific data. These methods concentrate on tumor size, invasion depth, and metastasis but fail to integrate essential insights from pathological images, radiological imaging, and clinical data, resulting in discrepancies between predictions and actual outcomes [6, 7, 8]. Key prognostic factors, including tumor budding and features of the tumor microenvironment such as immune cell density, can be identified through imaging modalities, but are not accounted for by current staging systems. Additionally, clinical reports, which include essential information on family history, disease progression, and laboratory markers, remain underutilized. This highlights a critical requirement for a multimodal prognostic framework that integrates pathological images, radiological imaging, and clinical reports [9, 10, 11]. Such an approach has the potential to provide more accurate risk assessments for survival rates and disease progression, thereby facilitating personalized treatment plans and optimized follow‐up strategies, ultimately enhancing patient outcomes and life quality.

Recent advancements in deep learning have positioned multimodal data fusion as a critical focus within medical data analysis [10, 11]. Unlike traditional approaches that depend on manual feature engineering, deep learning enables the automated extraction of critical features from diverse modalities, thereby demonstrating an unprecedented potential. However, the integration of pathology images, radiological images, and textual reports for accurate disease prognosis prediction remains challenging due to several unresolved issues.

The major challenge resides in the complexity of multimodal data and the heterogeneity across different modalities. Current research predominantly centers on single‐modality data, such as pathology images [1, 2, 3, 12, 13, 14, 15, 16], radiological images [17, 18, 19], or text data [20, 21, 22], as well as dual‐modality fusion, including the integration of pathology images with genomic data [9, 23], radiological images [24], or text [25, 26, 27]. Current unimodal architectures that leverage self‐attention mechanisms [1, 17, 28], as well as dual‐modality interaction models that utilize cross‐attention [9, 23, 24, 25], exhibit limitations in their capacity to effectively process three or more modalities. Furthermore, the inherent disparities in data attributes across different modalities further intensify these limitations [10, 11]. Pathology images, characterized by their ultra‐high resolution, are 2D data that necessitate segmentation into thousands of patches as a network input. Radiological images, such as CT scans, require 3D layer‐wise analysis, and textual reports demand semantic modeling to capture their content. These complexities, in conjunction with the scarcity of multimodal datasets, frequently impede effective end‐to‐end training and necessitate the utilization of advanced feature extraction techniques and sophisticated architectural designs.

Another significant challenge is the problem of missing modalities, which frequently occurs in clinical practice. Numerous existing models [9, 24, 25, 29, 30, 31, 32] assume the availability of complete data for at least some modalities during either training or testing phases, thereby constraining their practical applicability in clinical environment. Strategies such as late fusion of unimodal outputs [33], feature‐level aggregation [30, 31, 32, 34], or generating missing modalities from existing ones [35] have been proposed in previous works; however, each of these approaches is associated with notable limitations. Late fusion is limited in its ability to capture deep interactions between modalities, and its prediction accuracy can be influenced by biases inherent in unimodal predictions. Feature‐level aggregation is an overly simplistic approach that fails to integrate modality‐specific features. Moreover, the approach of the generation of missing modalities remains a highly complex and error‐prone process.

A third significant limitation is the insufficient interpretability of multimodal deep learning models. These models are difficult to interpret in terms of the clinical, immunological, or genomic patterns that underlie their predictions [11, 36, 37]. Although visualization approaches [1, 3, 9, 33, 36] such as heatmaps can highlight the regions of interest in pathology images, they frequently fall short in providing a comprehensive understanding of intermodal interactions and the underlying factors that contribute to prognosis. Addressing these interpretability challenges is critical for the development of robust models that can accurately integrate multimodal data, effectively manage missing modalities, and fully excavate the potential ability of multimodal medical data for prognostic prediction.

In this paper, we collected a multimodal dataset from 1,679 colorectal cancer patients across four large‐scale independent clinical centers. The dataset comprising pathological slides, radiological images, and clinical reports, which include molecular marker mutation data as well as other clinicopathological information. Using this dataset, we developed the **F**oundation Mode**L**‐Powered Multimod**A**l Deep Lea**R**ning model with Missing Modaliti**E**s, referred to as **FLARE**, a novel approach for enhancing prognostic prediction accuracy in colorectal cancer. FLARE leverages modality‐specific pre‐trained foundational models, a multi‐branch attention‐based architecture, and multiple strategies to address missing modalities and to enhance robustness and generalizability in the analysis of multimodal medical data.

The performance of FLARE was retrospectively evaluated utilizing the Concordance Index (C‐index) and Kaplan‐Meier analysis across an internal validation set as well as three external validation cohorts. It achieved the highest C‐index values across all cohorts in two tasks: Overall Survival (OS) and Progression‐Free Survival (PFS) prediction, demonstrating superior generalizability and predictive accuracy across multi‐center datasets. FLARE effectively performed risk stratification, categorizing patients into high‐risk and low‐risk groups. This finding highlights the clinical robustness and reliability of FLARE as a prediction tool. Furthermore, the superior performance of FLARE has been demonstrated, compared with traditional clinical models and current state‐of‐the‐art multimodal deep learning models across all validation cohorts. To enhance interpretability, FLARE was evaluated with respect to its emphasis on critical clinical factors, levels of immune infiltration, and gene pathways. The critical features identified by the model have been highlighted through heatmap visualizations of pathological slides, radiological images, and textual data, which can provide valuable insights into the decision‐making process of the model.

## 2 Results

### 2.1 Overall Design

**FIGURE 1:**
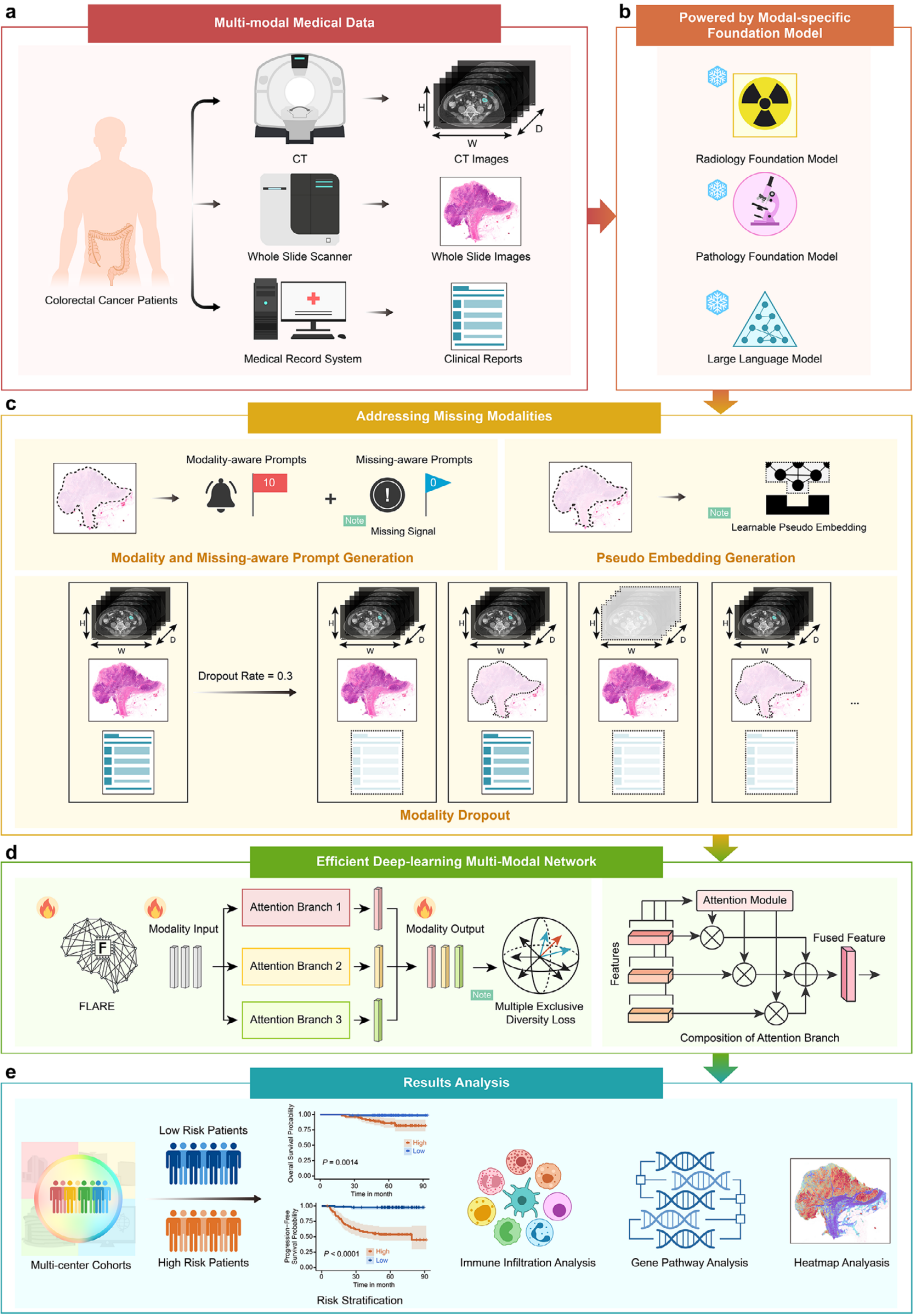
Overall design of the study. (a) This study utilized a multimodal dataset of colorectal cancer patients from four independent large clinical centers, encompassing pathological slides, radiological images, and clinical text reports, which include molecular marker mutation data as well as other clinicopathological information. (b) The proposed FLARE model achieves efficient feature extraction by incorporating modality‐specific pre‐trained foundational models. (c) To address the challenge of missing modalities, FLARE employs modality‐aware and missing‐aware prompts, learnable pseudo embeddings, and modality‐level data augmentation strategies. (d) FLARE employs an attention‐based multi‐branch exclusive modeling framework, which enhances feature diversity through an exclusive diversity loss function. (e) The performance of FLARE was comprehensively evaluated in an internal validation set and three external validation cohorts, using the C‐index and Kaplan‐Meier analysis for high‐risk and low‐risk stratification of patients. To improve the interpretability of FLARE's decision‐making process, we conducted statistical analysis focusing on pivotal clinical factors, the levels of immune infiltration, and gene pathway evaluations. Heatmap visualizations of pathological slides, radiological imaging, and text modalities highlighted the salient clinical features prioritized by the model.

Figure 1 illustrates the overall design and framework of this study. As depicted in Figure 1a, a multimodal dataset was collected from 1679 colorectal cancer patients across four independent clinical centers, including pathological slides, radiological imaging, and clinical text reports containing crucial molecular biomarker mutations and other clinicopathological data. The objective was to develop a robust multimodal deep learning model for accurate prognosis prediction in colorectal cancer, encompassing precise forecasting of survival rates and disease progression. As shown in Figure 1b–d, we proposed the FLARE model, which addressed the challenge of missing modalities using modality‐specific pre‐trained foundational models, a multi‐branch attention‐based framework, and various strategies for handling incomplete data. These approaches could enhance FLARE's robustness and generalizability across diverse datasets. As depicted in Figure 1e, FLARE was comprehensively evaluated using Kaplan‐Meier analysis and the C‐index across an internal validation set and three external validation cohorts. To improve the interpretability of FLARE's decision‐making process, we conducted statistical analysis focusing on pivotal clinical factors, the levels of immune infiltration, and gene pathway evaluations. Heatmap visualizations of pathological slides, radiological imaging, and text modalities highlighted the salient clinical features prioritized by the model, providing valuable insights into its predictive outcomes.

### 2.2 Multi‐Center and Multi‐Modal Dataset and Cohort Characteristics

**FIGURE 2:**
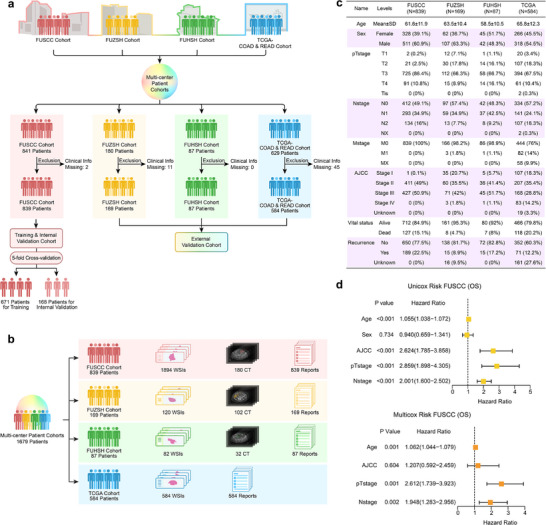
Composition of the multi‐center multimodal dataset and cohort statistical characteristics. (a) Enrollment of multi‐center data and dataset division. (b) Distribution and composition of multimodal data. (c) Distribution of statistical characteristics in the enrolled multi‐center dataset. (d) Results of univariate and multivariate Cox regression analysis on the training set FUSCC cohort.

This study retrospectively collected and analyzed a multimodal dataset of 1679 colorectal cancer patients from four independent clinical centers: Fudan University Shanghai Cancer Center (FUSCC), Fudan University Zhongshan Hospital (FUZSH), Fudan University Huashan Hospital (FUHSH), and the publicly available TCGA‐COAD&READ database. The distribution of patients across centers is shown in Figure 2a. Following rigorous screening by experts in pathology, radiology, and clinical medicine based on inclusion criteria (detailed in the Methods section), the final cohort comprised 839 patients from FUSCC, 169 from FUZSH, 87 from FUHSH, and 584 from TCGA‐COAD&READ.

The dataset includes whole‐slide pathological images, radiological CT scans, and clinical reports containing critical information, such as mutations in molecular biomarkers. Missing modalities are present for some patients, with distributions outlined in Figure 2b. Expert annotations of lesion areas were performed by specialists. Clinical reports provide patient history, pathology, radiology, and colonoscopy findings, with further details in the **Methods** and Supplementary Information.

The FUSCC cohort includes 1,894 H&E‐stained whole‐slide images (368 patients), CT scans (180 patients), and clinical reports (839 patients). The FUZSH cohort contains 120 H&E‐stained images (120 patients), CT scans (102 patients), and clinical reports (169 patients). The FUHSH cohort comprises 82 H&E‐stained images (75 patients), CT scans (32 patients), and clinical reports (87 patients). The TCGA‐COAD&READ cohort includes 584 H&E‐stained images and clinical reports (584 patients). Demographic and prognostic data, including AJCC/TNM staging, survival, and recurrence status, were collected from all patients, and their distributions are presented in Figure 2c and the additional details in Supplementary Information. The dataset was partitioned as follows: the FUSCC cohort was used for training and internal validation (five‐fold cross‐validation), while the FUZSH, FUHSH, and TCGA‐COAD&READ cohorts served as independent external validation sets.

### 2.3 Multimodal Deep Learning Model FLARE

**FIGURE 3:**
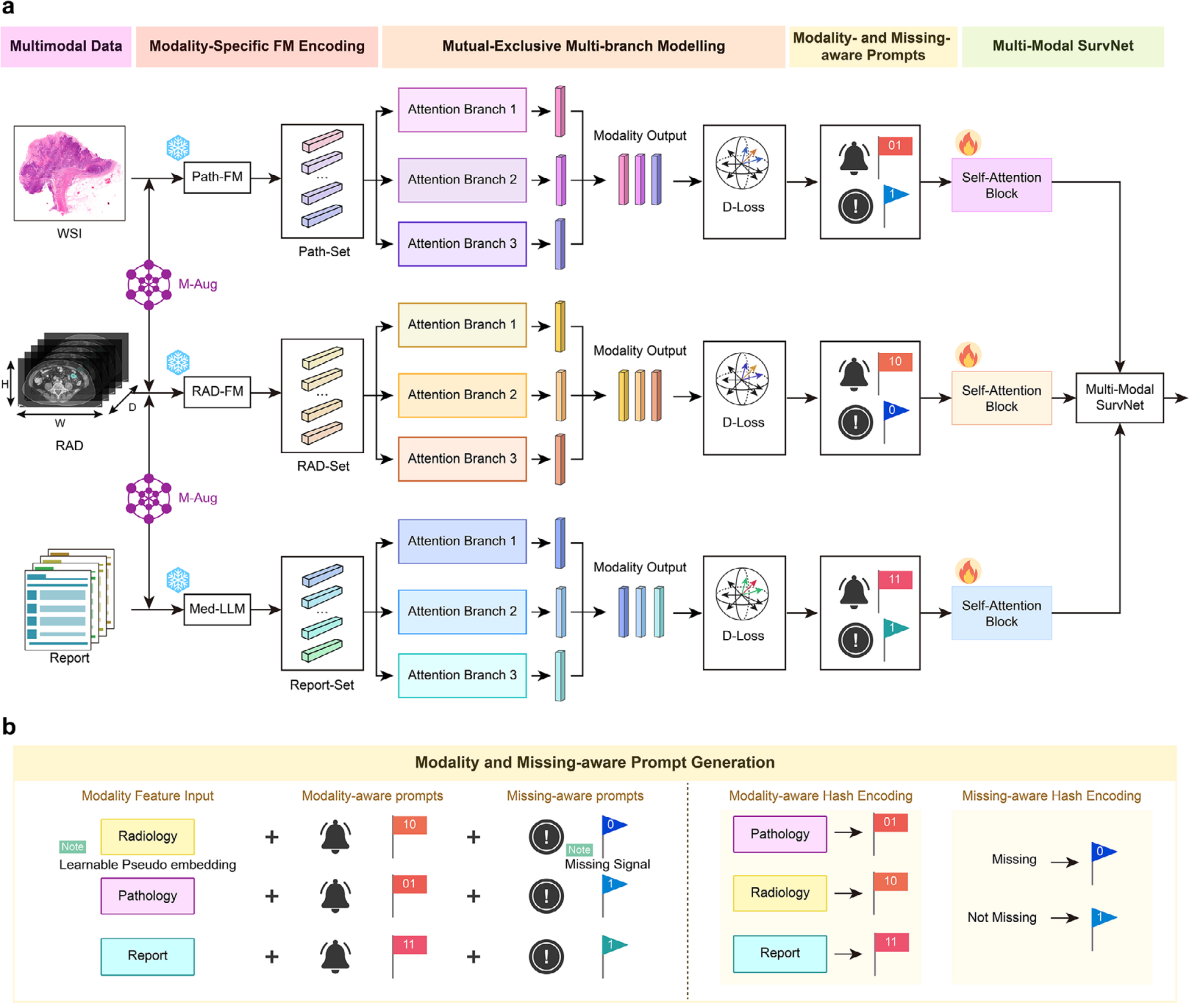
Framework of FLARE. (a) FLARE utilizes pre‐trained modality‐specific foundational models to pre‐encode features from pathological images, radiological images, and medical reports, and extracts mutually exclusive prognostic features through a multi‐branch attention mechanism. Simultaneously, a mutually exclusive diversity loss function is introduced to promote differentiated feature learning. Ultimately, precise patient prognosis evaluation is achieved through self‐attention modules and a multi‐modal prognostic risk prediction network. (b) To address the issue of missing modalities, FLARE employs modality‐aware and missing‐aware prompts and learnable pseudo‐embeddings.

As depicted in Figure 3a, FLARE is a multimodal deep learning framework driven by foundational models, specifically designed to tackle the challenge of missing modalities. The detailed exposition of the design and implementation is detailed in the Methods section. FLARE utilizes pre‐trained foundational models (FMs) for pathological images, radiological imaging, and clinical reports to efficiently encode modality‐specific features. These features are processed via a multi‐branch attention‐based framework that extracts prognosis‐relevant information from multiple perspectives by employing parallel attention mechanisms. To ensure diverse feature extraction, FLARE incorporates a specialized diversity loss function to promote differentiated feature learning across branches. The integrated features are processed through a self‐attention mechanism and subsequently fed into a fully connected multimodal prognostic risk prediction network to estimate the prognosis risk.

To address the issue of missing modalities, FLARE employs three novel techniques, as depicted in Figures 1c and 3b. First, modality‐aware and missing‐aware prompts are designed to encode the presence or absence of each modality within the features set, thereby facilitating the explicit identification of missing data and enhancing the optimization of multimodal integration. Second, learnable pseudo embeddings are employed to dynamically substitute for missing modality inputs, compensating for data deficiencies and maintaining model performance. Third, a modality‐level data augmentation strategy that randomly omits modalities during the training phase is utilized. This approach simulates the scenarios of missing data during testing, thus significantly enhancing the robustness of FLARE.

### 2.4 FLARE Achieves Superior Prognostic Performance

**FIGURE 4:**
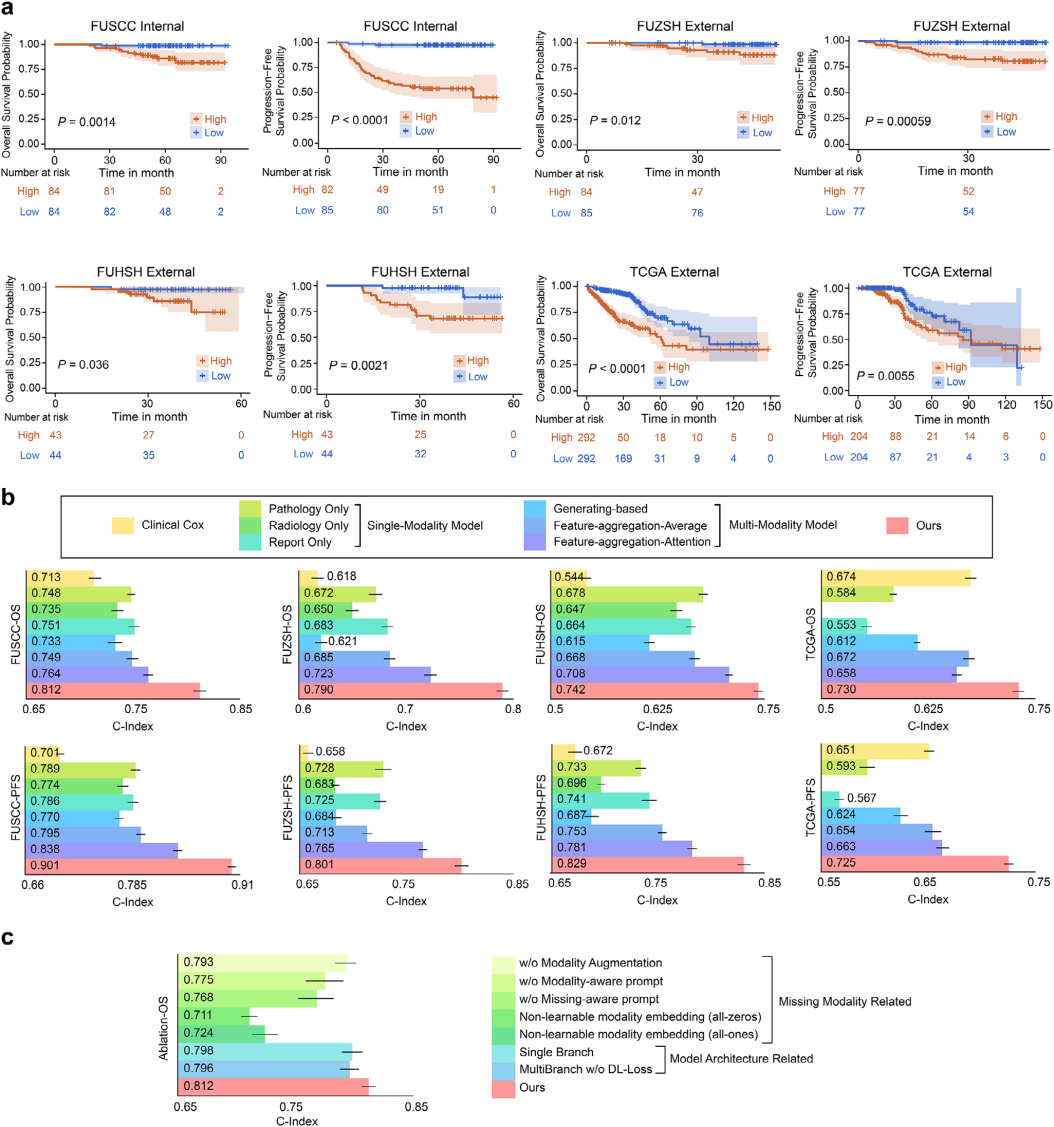
Performance evaluation of FLARE in patient prognosis prediction. (a) FLARE significantly stratifies high‐risk and low‐risk groups in four patient cohorts (OS and PFS) through KM analysis, and the statistical significance of its risk stratification is validated by the Log‐rank test. (b) FLARE significantly outperforms clinical models and other comparative deep‐learning models in C‐index results for OS and PFS prediction tasks across four cohorts. (c) Ablation studies in the FUSCC cohort for OS prediction demonstrate the effectiveness of FLARE's multimodal approach and individual components.

FLARE's prognostic prediction performance for OS and PFS was comprehensively evaluated across four cohorts using Kaplan‐Meier (KM) analysis and the concordance index (C‐index). KM analysis demonstrated FLARE's effectiveness in stratifying patients into high‐ and low‐risk groups, confirming its potential as an independent prognostic biomarker. C‐index quantified FLARE's capability to rank patients according to their risk levels. The results of KM analysis are presented in Figure 4a, and C‐index evaluations are illustrated in Figure 4b. Notably, radiological unimodal models were excluded for the TCGA‐COAD&READ cohort due to the lack of available radiological data.

#### 2.4.1 FLARE Enables Robust Risk Stratification

The capability of FLARE to stratify patients according to risk was validated using KM analysis for OS and PFS across all cohorts. The survival differences between high‐ and low‐risk groups were assessed using the Log‐rank test. Patients were stratified into high‐ and low‐risk groups according to the median of model‐predicted risk scores. As illustrated in Figure 4a, FLARE significantly differentiated between the two groups across all cohorts, with low‐risk patients exhibiting substantially improved OS and PFS outcomes.

For OS prediction, the Log‐rank test yielded statistically significant results across all cohorts: *p* = 0.0014 for the FUSCC cohort, *p *< 0.0001 for the FUZSH cohort, *p* = 0.036 for the FUHSH cohort, and *p* < 0.0001 for the TCGA cohort. For PFS prediction, FLARE also showed significant performance, with *p* < 0.0001 for the FUSCC cohort, *p* = 0.00059 for the FUZSH cohort, *p* = 0.0021 for the FUHSH cohort, and *p* = 0.0055 for the TCGA cohort. These results confirm FLARE's strong risk stratification capability across all cohorts, underscoring its reliability and clinical applicability.

#### 2.4.2 FLARE Exhibits Exceptional C‐Index Performance

As shown in Figure 4b, FLARE achieved a high C‐index across all cohorts for both OS and PFS predictions, highlighting its predictive robustness. For OS prediction, the model achieved a C‐index of 0.812 (95% CI: 0.806–0.818, *p* < 0.001) in the FUSCC cohort, 0.790 (95% CI: 0.784–0.795, *p* < 0.001) in the FUZSH cohort, 0.742 (95% CI: 0.736–0.747, *p* < 0.001) in the FUHSH cohort, and 0.730 (95% CI: 0.723–0.736, *p* < 0.001) in the TCGA cohort. For PFS prediction, FLARE achieved a C‐index of 0.901 (95% CI: 0.896–0.906, *p* < 0.001) in the FUSCC cohort, 0.801 (95% CI: 0.795–0.807, *p* < 0.001) in the FUZSH cohort, 0.829 (95% CI: 0.823–0.835, *p* < 0.001) in the FUHSH cohort, and 0.725 (95% CI: 0.720–0.730, *p* < 0.001) in the TCGA cohort.

These results exhibit FLARE's superior performance across both internal and external validation cohorts. Its ability to consistently deliver high C‐index values highlights its generalizability and predictive robustness across diverse patient populations, establishing FLARE as a dependable tool for prognosis prediction in colorectal cancer.

### 2.5 Comparative and Ablation Studies of FLARE

#### 2.5.1 FLARE Outperforms Clinical and Existing Deep Learning Models

To comprehensively evaluate FLARE's performance, we conducted a comparative analysis against both traditional clinical models and state‐of‐the‐art multimodal deep learning methods. The clinical baseline model was developed using Cox regression, incorporating AJCC/TNM staging and other crucial clinical factors (e.g., age), which were selected via univariate Cox regression analysis in the FUSCC training cohort, as illustrated in Figure 2d. This model was trained on the FUSCC cohort and validated on external cohorts. For multimodal deep learning baseline models, we included three mainstream approaches: generative models (generating missed modalities using existing modalities before fusion), feature averaging models (simple fusion by averaging modality features), and attention aggregation models (weighted modality fusion using attention). The details of these baseline models are provided in the Supplementary Information.

As shown in Figure 4b, FLARE outperformed all baseline models across four cohorts in both OS and PFS prediction. For OS prediction, FLARE achieved a higher C‐index across all cohorts, with improvements over the Cox model ranging from 8.3% to 36.4% and over the best deep learning baseline model ranging from 4.8% to 9.3%. Similarly, for PFS prediction, FLARE consistently demonstrated significant performance gains, with C‐index improvements of 11.4% to 28.5% over the Cox model and 4.7% to 9.4% over the best deep learning baseline. These results underscore FLARE's exceptional generalization capability and predictive robustness in survival prediction tasks.

#### 2.5.2 FLARE Outperforms Unimodal Baselines

To assess the contribution of multimodal integration, we compared FLARE's performance with unimodal models using pathological images, radiological images, and clinical reports separately. Across all cohorts, multimodal models outperformed unimodal counterparts for both OS and PFS predictions.

For OS prediction, unimodal models demonstrated varying performance, with pathological images generally achieving the best results, followed by clinical reports, while radiological imaging often lagged. For example, in the FUSCC cohort, the C‐index was 0.748 for pathology, 0.751 for clinical text, and 0.735 for radiology. In contrast, FLARE achieved a significantly higher C‐index by effectively integrating features across modalities.

For PFS prediction, similar trends were observed. In the FUZSH cohort, the pathological modality achieved a C‐index of 0.728, clinical text 0.725, and radiology 0.683, while FLARE surpassed all unimodal models by a substantial margin. These results highlight the superiority of FLARE's multimodal fusion strategy over individual modalities.

#### 2.5.3 Ablation Studies Validate the Effectiveness of the Components of FLARE

To evaluate the contributions of individual components in FLARE, we performed ablation experiments focused on modality enhancements, prompt mechanisms, and model structure. Modality enhancement and prompts (modality‐aware and missing‐aware) were critical for performance. Removing modality enhancement reduced the C‐index from 0.812 to 0.793. Omitting modality‐aware and missing‐aware prompts further reduced the C‐index to 0.775 and 0.768, respectively. Replacing learnable modality embeddings with fixed zero or one vectors degraded performance to 0.711 and 0.724, respectively. The multi‐branch structure and diversity loss function (DL‐Loss) also played pivotal roles. Changing the multi‐branch framework to a single‐branch model reduced the C‐index to 0.798, while removing the DL‐Loss resulted in a C‐index of 0.796.

These findings demonstrate that each component of FLARE, including modality‐specific enhancements, prompt mechanisms, and the multi‐branch structure, contributes significantly to its performance. Together, they highlight the design rationale behind FLARE and its efficacy as a multimodal deep learning model for medical prediction tasks.

### 2.6 FLARE's Interpretability with Clinical Insights

#### 2.6.1 Heatmap Analysis of Pathology, Radiology, and Clinical Report

**FIGURE 5:**
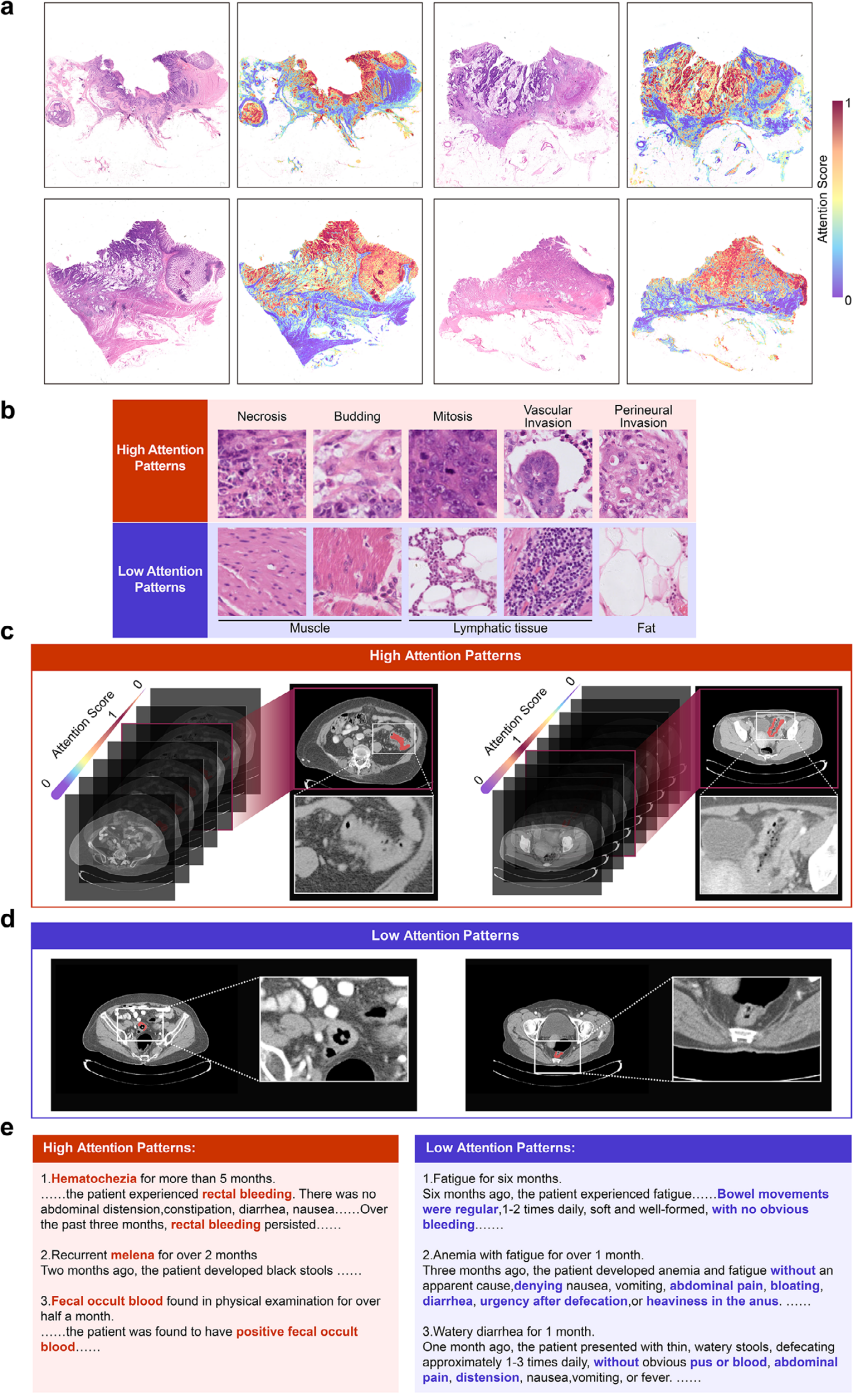
Modal interpretability analysis of FLARE in histopathology, radiological imaging, and clinical reports. (a) FLARE visualizes the attention scores on histopathology WSI through heatmaps. Darker red regions indicate higher attention weights, while darker blue regions represent lower attention weights. (b) Typical patterns of high‐attention and low‐attention regions identified by FLARE in histopathology image modality. (c) Typical patterns of high‐attention regions identified by FLARE in radiological CT imaging modality. (d) Typical patterns of low‐attention regions identified by FLARE in radiological CT imaging modality. (e) Typical patterns of high‐attention and low‐attention regions identified by FLARE in clinical reports.

FLARE interprets prognosis by quantifying attention scores across modalities—pathological images, radiological CT scans, and clinical reports —highlighting key prognostic areas and patterns. In pathological images, FLARE focuses on tumor extent and growth patterns, particularly in regions showing invasive growth into the serosa or subserosa and surrounding cancerous nodules, as is shown in Figure 5a. High‐risk patients display a higher proportion of patches with necrosis, budding, mitosis, vascular invasion, and perineural invasion—hallmarks of poor prognosis—while less attention is given to irrelevant areas like smooth muscle or adipose tissue, as is shown in Figure 5b.

For radiological CT scans, FLARE identifies critical high‐risk areas, including tumor invasion into the muscularis and surrounding fat spaces, as well as regional lymph node enlargement, as shown in Figure 5c. Low‐risk layers, where the tumor is confined to the lumen with clear fat spaces, are also identified as prognostically favorable, as is shown in Figure 5d. In clinical reports, FLARE prioritizes terms such as “hematochezia” and “fecal occult blood,” which are strongly associated with prognosis, as is shown in Figure 5e.

#### 2.6.2 Clinical Factor Analysis

**FIGURE 6:**
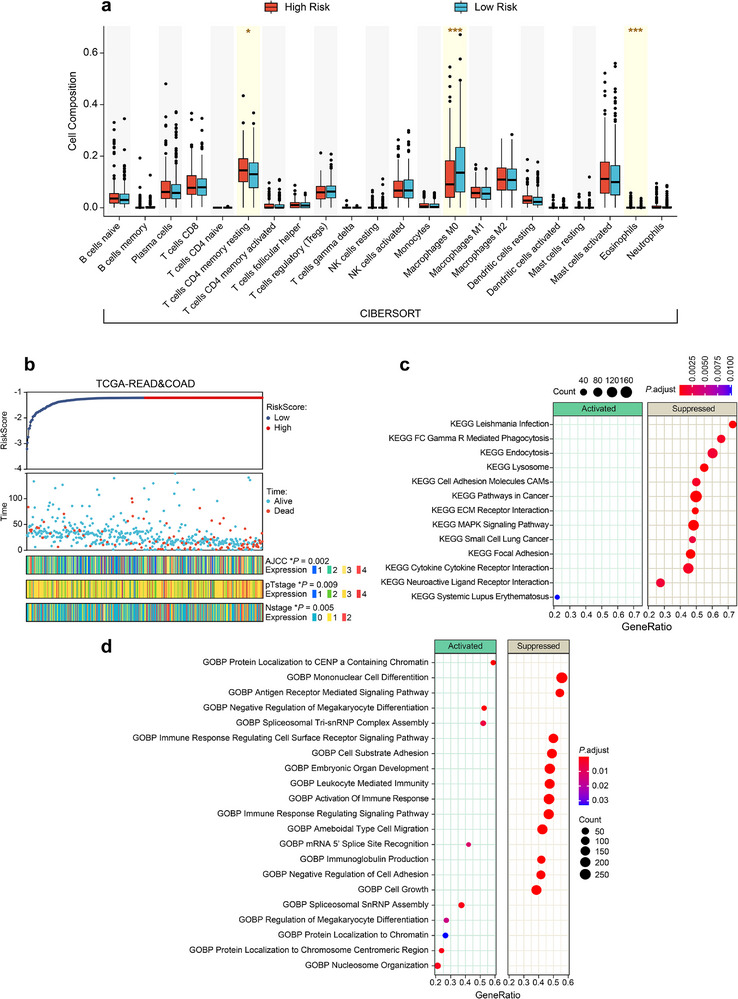
Interpretability analysis of important clinical factors, immune cell microenvironment, and gene pathways of FLARE in the TCGA‐COAD&READ cohort. (a) Statistical plot of the immune cell microenvironment in the high‐risk and low‐risk groups stratified by FLARE using the CIBERSORT method. (b) Statistical plot of important clinical factors (AJCC, pTstage, Nstage) in the high‐risk and low‐risk groups stratified by FLARE using statistical analysis methods. FLARE‐stratified low‐ and high‐risk groups exhibit significantly different distributions of AJCC stage, pT stage, and N stage. (c) Bubble plot of GSEA‐KEGG gene pathways that are significantly activated or suppressed when comparing the FLARE‐stratified high‐risk and low‐risk groups. (d) Bubble plot of GSEA‐GO gene pathways that are significantly activated or suppressed when comparing the FLARE‐stratified high‐risk and low‐risk groups.

To understand FLARE's stratification of high‐ and low‐risk groups, crucial clinical factors including AJCC staging, pTstage, and Nstage were analyzed in the TCGA‐COAD&READ cohort. Heatmap visualization highlights significant differences in these factors between risk groups, as is shown in Figure 6b, reinforcing their clinical relevance in FLARE's predictions. As shown in Figure 6b, we use the FLARE model trained on the internal FUSCC cohort to predict risk scores for all patients in the external TCGA‐COAD and READ cohorts. Based on the median predicted risk score, this cohort is divided into a FLARE stratified low‐risk group and a FLARE stratified high‐risk group. The top panel of Figure 6b shows the distribution of predicted risk scores for all patients, sorted by increasing risk, with the low‐risk group in dark blue and the high‐risk group in dark red. The middle panel displays the corresponding survival time and survival status for each patient, where light blue indicates alive and light red indicates deceased.

To examine the clinical interpretability of FLARE, we further compare key staging variables between the FLARE predicted high risk and low‐risk groups. The bottom panel of Figure 6b visualizes the distributions of AJCC stage, p‐T stage, and N stage for all patients. For each patient, the stage category is encoded by a color scale that ranges from cooler colors to warmer colors to represent discrete stage values. We perform formal statistical tests comparing the high‐risk and low‐risk groups for each staging variable. The results show significant differences in AJCC stage (*p* = 0.002), p‐T stage (*p* = 0.009), and N stage (*p* = 0.005). Importantly, FLARE is trained only on pathology whole slide images, CT images, and clinical narrative reports, and does not use these staging variables as input. These findings indicate that FLARE can automatically learn and use prognostically relevant staging information from raw imaging and textual data, in a way that is consistent with the key factors considered by clinical experts.

#### 2.6.3 Immune Microenvironment Analysis

The biological basis of FLARE's prognostic predictions was explored through immune infiltration analysis using the CIBERSORT method [38, 39] on the TCGA‐COAD&READ cohort. The proportions of 22 immune cell types were compared between high‐ and low‐risk groups. Significant differences (*p* < 0.05) were observed in resting CD4 memory T cells, M0 macrophages, and eosinophils, as shown in Figure 6a, suggesting that immune cell composition plays a critical role in FLARE's risk stratification. These findings emphasize the link between immune microenvironment variations and prognosis.

#### 2.6.4 Gene Pathway Enrichment Analysis

To investigate underlying molecular mechanisms, gene pathway enrichment analysis was performed using GSEA [40] on the KEGG [41] and GO [42] databases in the TCGA‐COAD&READ cohort. In Figure 6c,d, the pathway analyses are based on a differential expression contrast of FLARE‐stratified high‐risk versus low‐risk patients. Pathways shown in the “Activated” panel correspond to gene sets enriched among genes that are upregulated in the high‐risk group relative to the low‐risk group. In contrast, pathways in the “Suppressed” panel are enriched among genes that are downregulated in the high‐risk group and relatively more active in the low‐risk group, thereby highlighting biological programs that distinguish the two risk groups and are more preserved in the low‐risk subgroup.

For GSEA of KEGG pathways (Figure 6c), FLARE identifies 13 significantly altered pathways. Most of these, including immune‐related pathways such as FC Gamma R‐mediated Phagocytosis and Cytokine Receptor Interaction, are suppressed in the FLARE‐predicted high‐risk group and relatively more active in the low‐risk group, whereas the Systemic Lupus Erythematosus pathway shows the opposite pattern.

For GSEA of GO biological processes (Figure 6d), FLARE identifies 21 significantly different pathways, with prominent changes in chromatin regulation and immune response. The high‐risk group shows activation of chromatin‐related pathways, such as Nucleosome Organization and Protein Localization to Chromatin, whereas immune‐related pathways, including Activation of Immune Response and Leukocyte‐Mediated Immunity, are suppressed in the high‐risk group and relatively preserved in the low‐risk group. Consistent with this, most significantly altered pathways fall into the “Suppressed” category. These results suggest that FLARE partly distinguishes high‐ and low‐risk patients by recognizing patterns of pathway suppression, particularly in immune‐related programs, thereby providing a biologically interpretable basis for its risk predictions in the context of tumor progression and immune regulation.

## 3 Conclusions

In this paper, we introduce FLARE, an innovative multimodal deep learning model that demonstrates exceptional performance in prognostic prediction for colorectal cancer patients. Although considerable advancements have been achieved in single‐modality research, effective cancer prognosis necessitates the integration of histopathology, radiology imaging, and clinical data [9, 10, 11, 33, 35]. Multimodal models have the capability to integrate a wide range of medical information, thereby emulating the comprehensive diagnostic and therapeutic approach employed by clinicians. Nonetheless, the challenges, including effective feature extraction, network optimization, and missing modalities issues, have constrained the advancement of multimodal deep learning models. The colorectal cancer dataset established in this study represents the largest multi‐center, multimodal dataset to date, encompassing pathology, radiology, and textual reports. The FLARE proposed in this study has achieved a superior prognostic performance across external validation cohorts from multiple independent medical centers, while it has generalizability and applicability across a wide range of clinical settings. The capability of FLARE to efficiently process these diverse data types has been demonstrated. Accordingly, compared with previous works, FLARE provides more precise prognostic evaluations, offering a practical and innovative approach for clinical decision‐making in colorectal cancer.

Recent advancements in medical foundation models have underscored their potential [26, 27, 43, 44, 45, 46, 47]; however, the application of these models to specific clinical challenges remains underexplored. The significant heterogeneity of medical data across different modalities poses substantial challenges for direct end‐to‐end training on raw data, especially when working with limited multimodal datasets. This frequently results in convergence issues and inefficient feature fusion. FLARE tackles these challenges by leveraging modality‐specific foundational models that have been pre‐trained on extensive datasets, thereby facilitating efficient feature extraction from pathology, radiology, and textual reports. This pre‐encoding strategy mitigates the complexity of training on limited datasets by ensuring that essential features are captured at an early stage, thereby enhancing both model performance and training efficiency. FLARE also introduces a novel perspective in foundation model research by concentrating on specific clinical challenges. This approach not only highlights the potential of foundation models to address complex medical problems but also provides a roadmap for their practical application in clinical settings. By integrating foundational research with practical clinical requirements, FLARE presents a promising framework that facilitates advancements in multimodal medical analytics and improves patient outcomes.

In addition, compared to previous multimodal deep learning models, FLARE offers several notable advantages in addressing the issue of missing modalities. By incorporating modality‐ and missing‐aware prompts along with a learnable pseudo‐embedding mechanism, FLARE effectively addresses the feature gap caused by missing modalities, thereby mitigating performance degradation. Moreover, its modality‐level data augmentation strategy enhances the robustness in the training phase and facilitates dynamic adaptation during testing. Unlike preceding straightforward approaches, such as late fusion [33], feature averaging [34], or modality generation [35], FLARE effectively captures deep interactions among available modalities while dynamically adjusting the impact of missing modalities on predictions. This innovative approach enables accurate prognostic risk assessment, making FLARE particularly suitable for clinical applications where multimodal data may be incomplete due to cost constraints, technical limitations, or time restrictions. In single‐modality analyses, pathology and clinical report modalities often outperform radiology modalities. Pathology, as the visual gold standard, provides critical insights into the tumor microenvironment, while textual reports contain rich, clinically curated information about tumors, patient history, and molecular characteristics. These advantages may explain their superior performance; however, dataset‐specific biases or differences in prognostic information across modalities may also contribute to this observation.

The clinical interpretability of FLARE has also been presented via multiple dimensions, including heatmap visualizations, crucial clinical factor analysis, immune infiltration, and gene pathway enrichment. Heatmaps of pathology, radiology, and textual reports reveal that FLARE effectively identifies prognostically relevant regions, aligning closely with clinical practice in cancer diagnosis and treatment. FLARE's capability to stratify between high‐ and low‐risk groups based on AJCC/TNM staging underscores its dependence on well‐established clinical indicators, thereby enhancing its credibility in clinical settings. Immune infiltration analysis suggests that high‐risk patients may exhibit an immunosuppressive microenvironment. The high‐risk group shows a higher proportion of resting CD4 memory T cells, which lack effective antitumor activity due to inhibitory signals such as TGF‐β, IL‐10, or PD‐L1 expression [48, 49, 50]. Additionally, a lower proportion of M0 macrophages in the high‐risk group reflects an imbalance in pro‐inflammatory and anti‐inflammatory signals, promoting tumor immune evasion [51, 52, 53]. Conversely, elevated eosinophil levels in high‐risk patients suggest chronic inflammation, a known driver of tumor progression [54, 55, 56]. Gene pathway enrichment analysis corroborates these findings, revealing suppression of immune‐related pathways such as FC Gamma R‐Mediated Phagocytosis, Lysosome, and Cytokine‐Cytokine Receptor Interaction in the high‐risk group. These alterations suggest impaired immune clearance and weakened intercellular signaling, undermining antitumor immunity [57, 58]. Further suppression of pathways related to immune response regulation and activation highlights the role of immune evasion in poor prognosis [59, 60]. These findings illustrate FLARE's capacity to distinguish the biological underpinnings of high‐ and low‐risk groups, offering valuable insights into tumor progression and immune dynamics. Future research could incorporate multi‐omics and proteomics data to further validate these pathways and refine their clinical relevance.

The flexibility and modular architecture of FLARE make it an ideal platform for multimodal data analysis, enabling robust interdisciplinary collaboration. By integrating bioinformatics, clinical medicine, and deep learning techniques, FLARE's scalable framework enables adaptation to various cancer types and diagnostic tasks by retraining on specific disease cohorts, enhancing its applicability across oncology and diverse medical fields. Furthermore, FLARE's potential capability to incorporate real‐time data from wearable devices and remote monitoring tools, such as patient activity levels and physiological responses, will significantly enhance its multimodal functionalities. This adaptability establishes FLARE as a promising tool in the field of precision medicine, supporting comprehensive disease management and treatment optimization.

While acknowledging the contributions outlined above, this work has several limitations that point to clear directions for future research. First, FLARE was developed and evaluated in a retrospective multicenter setting, with training on the internal cohort and external validation in three other independent cohorts. Although this design goes beyond single‐center internal validation and provides reasonably strong evidence of robustness and generalizability, it cannot substitute for prospective evaluation embedded in real‐world clinical workflows. Second, potential distribution biases may persist because the cohort composition and treatment patterns can differ between early and advanced disease stages across centers, and our current analyses may not fully capture these effects. Third, while the dataset integrates pathology, radiology, clinical text, and key molecular markers, it does not include raw genomic sequencing data, which could further enhance biological interpretability and predictive accuracy. Finally, FLARE has so far been trained and validated only in colorectal cancer, and its utility in other tumor types or clinical scenarios needs further verification. Future work will focus on more extensive multicenter validation with broader and more diverse patient populations, integration of additional modalities such as high‐dimensional genomic data, and systematic evaluation of FLARE across different cancer types and clinical applications. In parallel, we plan to conduct stepwise prospective studies, beginning with “silent” deployment where FLARE runs alongside routine care without influencing treatment decisions, followed by carefully designed interventional trials to rigorously assess its impact on clinical decision making and long‐term patient outcomes.

In conclusion, FLARE integrates deep learning methodologies with multimodal data, offering a robust and versatile framework for prognostic prediction in colorectal cancer. It represents a significant advancement in personalized treatment strategies by combining pathology images, radiological imaging, and clinical reports through deep fusion techniques. FLARE demonstrates superior performance in synergistic multimodal analysis by effectively addressing challenges associated with missing modalities, enhancing predictive robustness, and optimizing patient management. Its modular design and adaptable architecture provide a strong foundation for future multimodal deep learning research and clinical applications, particularly in oncology and precision medicine. By advancing multimodal deep learning, FLARE facilitates improved patient outcomes and supports the development of innovative medical solutions.

## 4 Experimental Section

### 4.1 Data Ethics, Collection, and Inclusion Criteria

This study retrospectively collected and analyzed a multimodal dataset from 1,679 colorectal cancer patients across four independent large clinical centers: Fudan University Shanghai Cancer Center (FUSCC), Fudan University Zhongshan Hospital (FUZSH), Fudan University Huashan Hospital (FUHSH), and the publicly available TCGA‐COAD&READ database. The composition of data from each center is shown in Figure 2a. A total of 1,095 patients from FUSCC, FUZSH, and FUHSH, who underwent radical surgical resection for colorectal adenocarcinoma between January 2007 and January 2023, were included. Inclusion criteria were: diagnosis of colorectal adenocarcinoma confirmed by surgical resection, no prior treatments or other tumor histories, complete follow‐up and survival data (OS and PFS), and availability of postoperative tumor pathology H&E slides. Informed consent was obtained for all samples, and the study was approved by the Ethics Committee of the World Health Organization Collaborating Center for Human Reproductive Research (approval number: 2105235‐26).

Follow‐up data were collected through outpatient visits or phone calls from the cancer registry, including information on tumor recurrence, progression, and mortality. Recurrence and metastasis were monitored via routine CT or MRI scans at 6 months post‐surgery, followed by annual scans. OS was defined as the time from diagnosis to death or the end of the follow‐up period, while PFS was defined as the time from treatment initiation to disease progression, recurrence, or death.

### 4.2 Data Preprocessing

Pathological slides from enrolled patients were collected, and two senior pathologists independently reviewed the H&E‐stained slides, selecting regions from the most representative areas of the tumors. The slides were digitized using the Jiangfeng KF‐PRO‐005 scanner (Ningbo Jiangfeng Bioinformatics Technology Co., Ltd.) at 40x magnification, ensuring high‐quality whole‐slide images (WSIs) for each patient. A pathologist then analyzed the tumor regions within the WSIs and interactively marked regions of interest (ROIs).

Preoperative enhanced CT images were collected and re‐evaluated by radiologists. Tumor segmentation was performed on the arterial phase of CT images using ITK‐SNAP (version 4.0.2; http://www.itsnap.org).

Clinical data were retrieved from hospital medical records, including tumor location, grade, TNM stage, histological classification, vascular invasion, perineural invasion, circumferential resection margin involvement, and mutations of key molecular biomarkers, all of which were confirmed by clinical doctors. Clinical reports from the three hospitals were standardized by manually reviewing and harmonizing pathology, radiology, and clinical reports to eliminate discrepancies due to differing formats. Collected clinicopathological data included age, gender, tumor location, grade, TNM stage, histological classification, vascular invasion, perineural invasion, circumferential resection margin involvement, and molecular test results for KRAS, NRAS, BRAF, MMR, and MSI.

### 4.3 Multimodal Deep Learning Model FLARE

#### 4.3.1 FLARE Achieves Modality Preprocessing and Feature Encoding Through Modality‐specific Foundational Models

As shown in Figure 3a, FLARE employs pre‐trained modality‐specific foundational models (FMs) to efficiently pre‐encode features from multimodal data, such as pathological slides, radiological imaging, and clinical reports. Detailed descriptions of the modality‐specific foundational models can be found in the supplementary materials.

Preprocessing and encoding of the pathological modality (WSI). For pathological slides, FLARE uses the pre‐trained pathology vision‐language model PLIP [43] to pre‐encode visual features. Specifically, the whole slide image (WSI) is first divided into *n_W_* 224 × 224 image patches at a 20x magnification, based on the typical tumor regions annotated by pathology experts. Subsequently, the image encoder of the PLIP extracts a 1 × 512‐dimensional visual feature vector for each image patch, generating a corresponding *n_W_* ×512‐dimensional feature vector set. If a patient contains multiple WSIs, these feature vectors are concatenated to form an extended pathological feature vector set, denoted as $F_{i}^{W}\in R^{N_{i}^{W}\times 512}$, where $N_{i}^{W}$ represents the total number of image patches across all WSIs for patient *i*.

Preprocessing and encoding of the radiological imaging modality (CT). For radiological CT images, FLARE uses the pre‐trained radiological imaging foundational model MedSAM [61] to pre‐encode visual features. The specific steps include: based on the typical tumor regions annotated by radiological experts, the image encoder of the MedSAM and position‐based prompt encoder are used to extract layer‐by‐layer 512‐dimensional visual feature vectors from *n_R_* 2D layers, generating a set of radiological feature vectors containing both positional and visual information, denoted as $F_{i}^{R}\in R^{N_{i}^{R}\times 512}$, where $N_{i}^{R}$ represents the total number of 2D image layers for patient *i*.

Preprocessing and encoding of clinical text reports. For all medical reports (such as pathology, radiology, medical history, and colonoscopy reports), FLARE uses the pre‐trained large language model BioLinkBERT‐large [62] to extract text features. Specifically, the text encoder within the BioLinkBERT‐large is used to extract features from each type of report, generating corresponding feature vector sets. If a patient contains multiple report sections, these feature vectors are concatenated to form an extended text report feature vector set, denoted as $F_{i}^{T}\in R^{N_{i}^{T}\times 1024}$, where $N_{i}^{T}$ represents the total number of report sections for patient *i*.

#### 4.3.2 FLARE Introduces an Attention‐Based, Multi‐Branch, Mutually Exclusive Modeling Framework

Building upon the important multimodal features extracted from the foundational models, FLARE introduces a multi‐branch, mutually exclusive modeling framework based on the attention mechanism. This framework extracts prognostically relevant features from multiple perspectives through parallel attention branches. To ensure maximal diversity in the features extracted by different branches, FLARE incorporates a mutually exclusive diversity loss function, enhancing the differentiated feature learning ability between branches. Specifically, as shown in Figure 3a, for a patient's pathological feature vector set $F_{i}^{W}$, radiological feature vector set $F_{i}^{R}$ and text report feature vector set $F_{i}^{T}$, we establish three attention‐based intra‐modality feature aggregation branches, yielding three sets of aggregated features: pathological modality aggregation features $Z_{i,(m)}^{W}$, radiological modality aggregation features $Z_{i,(m)}^{R}$, and text modality aggregation features $\;Z_{i,(m)}^{T}$, where m = {1,2,3}.

For example, for the pathological feature vector set $F_{i}^{W}$, we input it into three attention aggregation networks with the same structure but different parameters:

(1) $$Z_{i,\left(m\right)}^{W}=\sum_{j=1}^{N_{i}^{W}}\;a_{i,j,\left(m\right)}^{W}F_{i,j}^{W},m=\left\{0,1,2\right\}$$

(2) $$a_{i,j,\left(m\right)}^{W}=\frac{\exp\left\{w_{\left(m\right)}^{⊤}\tanh\left(V_{\left(m\right)}F_{i,j}^{W^{⊤}}\right)\right\}}{\sum_{j=1}^{N_{i}^{W}}\exp\left\{w_{\left(m\right)}^{⊤}\tanh\left(V_{\left(m\right)}F_{i,j}^{W^{⊤}}\right)\right\}}$$

where$\;F_{i,j}^{W}$ represents the feature vector of each patch in the vector set $F_{i}^{W}$; $a_{i,j,(m)}^{W}$ is the attention weight predicted by the self‐attention network, determined by the learnable parameters *w*_(*m*)_ and *V*_(*m*)_, reflecting the contribution of each instance in the aggregation process.

Building upon this, we further introduce three sets of exclusive diversity loss functions, each constraining the maximization of parameter differences within the three attention aggregation networks generating $Z_{i,(m)}^{W}$, $Z_{i,(m)}^{R}$, and $Z_{i,(m)}^{T}$, thereby forcing the network to learn features strongly correlated with prognosis from different perspectives within each modality. Taking $Z_{i,(m)}^{W}$ as an example, the exclusivity diversity loss function $L_{\mathrm{diff}\;}$ is minimized to maximize the distance between each pair of features, thereby enhancing the discriminative power of the features. The specific form is as follows:

(3) $$L_{\mathrm{diff}\;}=-\frac{1}{3}\left(\mathrm{Dist}\left(Z_{i,\left(0\right)}^{W},Z_{i,\left(1\right)}^{W}\right)+\mathrm{Dist}\left(Z_{i,\left(0\right)}^{W},Z_{i,\left(2\right)}^{W}\right)+\mathrm{Dist}\left(Z_{i,\left(1\right)}^{W},Z_{i,\left(2\right)}^{W}\right)\right)$$

where the cosine distance is used as the metric function:

(4) $$\mathrm{Dis}t_{\cos}\left(Z_{i,\left(m\right)}^{W},Z_{i,\left(n\right)}^{W}\right)=1-\frac{\langle Z_{i,\left(m\right)}^{W},Z_{i,\left(n\right)}^{W}\rangle }{∥Z_{i,\left(m\right)}^{W}∥∥Z_{i,\left(n\right)}^{W}∥}$$

this yields:

(5) $$L_{diff}=-\frac{1}{3}\sum_{m\neq n}\left(1-\frac{\langle Z_{i,\left(m\right)}^{W},Z_{i,\left(n\right)}^{W}\rangle }{∥Z_{i,\left(m\right)}^{W}∥∥Z_{i,\left(n\right)}^{W}∥}\right)$$

#### 4.3.3 FLARE Addresses Modality Missingness Through Prompt Information, Pseudo Embeddings, and Data Augmentation

As shown in Figure 1c, to address the issue of missing modalities, FLARE incorporates three innovative techniques: modality‐aware and missing‐aware prompts, learnable pseudo embeddings, and modality‐level data augmentation strategies. These methods collectively enhance the model's robustness in scenarios where modalities are missing, enabling more reliable performance even with incomplete data.

Modality‐aware and missing‐aware prompts. FLARE introduces modality‐aware and missing‐aware prompts, embedding the modality status into the feature representation, enabling the model to explicitly recognize the presence or absence of each modality, thereby optimizing the integration of multimodal information. Specifically, as shown in Figure 3b, we assign a unique modality‐aware prompt for each patient's modality data. For instance, the hash encoding for the pathology modality is “01,” for the radiological imaging modality is “10,” and for the text report modality is “11.” Additionally, we assign missing‐aware prompts for the presence or absence of each modality; for example, when the imaging modality is missing, it is encoded as “0,” and when present, it is encoded as “1.” Each modality for every patient contains two types of prompts (modality‐aware and missing‐aware), which are concatenated in encoded form to the modality features, resulting in a new feature representation. In this way, FLARE can dynamically sense the integrity of modalities when processing multimodal data, enhancing the model's adaptability to missing modalities. Mathematically, based on the true modality status, we concatenate the modality‐aware prompt encoding φ_*mod*_ and the missing‐aware prompt encoding φ_*miss*_ to each aggregated modality vector $Z_{i,(m)}^{W}$, $Z_{i,(m)}^{R}$, and $Z_{i,(m)}^{T}$, resulting in the prompt‐enhanced modality vectors $H_{i,(m)}^{W}$, $H_{i,(m)}^{R}$, and $H_{i,(m)}^{T}$.

(6) $$\begin{array}{c} H_{i,\left(m\right)}^{W}=Concat\left(Z_{i,\left(m\right)}^{W},\varphi_{\mathrm{mod}}^{W},\varphi_{\mathrm{miss}}^{W}\right)\\ \;H_{i,\left(m\right)}^{R}=Concat\left(Z_{i,\left(m\right)}^{R},\varphi_{\mathrm{mod}}^{R},\varphi_{\mathrm{miss}}^{R}\right)\\ \;H_{i,\left(m\right)}^{T}=Concat\left(Z_{i,\left(m\right)}^{T},\varphi_{\mathrm{mod}}^{T},\varphi_{\mathrm{miss}}^{T}\right) \end{array}$$

Learnable pseudo embedding. To address missing modalities, FLARE introduces the learnable pseudo‐embedding technique. A pseudo embedding is a dynamically optimized vector that learns compensatory features for the missing modality during model training, mitigating the impact of information loss. As shown in Figure 3b, when a modality is missing for a patient, FLARE generates a learnable pseudo embedding vector for that modality and uses it as the input feature. This mechanism effectively compensates for the missing information, allowing the model to maintain stable predictive performance.

Modality‐level data augmentation strategy. To improve the model's robustness against modality missingness, FLARE employs a modality‐level data augmentation strategy. During training, modalities are randomly dropped with a certain probability to simulate missing modalities during testing. As shown in Figure 1c, assuming all three modalities are available for a patient, each modality is randomly dropped with a 30% probability at the start of each training iteration, creating a new combination of available modalities. To ensure the integrity of multimodal information, we require that at least one valid modality remains for each patient after each augmentation. This strategy enables FLARE to effectively handle modality missingness during training, enhancing its robustness during testing.

#### 4.3.4 The Prognostic Risk Prediction Network and Loss Function of FLARE

As shown in Figure 3a, after obtaining the prompt‐modified modality vectors $H_{i,(m)}^{W}$, $H_{i,(m)}^{R}$, and $H_{i,(m)}^{T}$, we establish three self‐attention modules to further process these modality features. The interacted modality vectors are concatenated and input into a multimodal prognostic risk prediction network (Multi‐Modal SurvNet), composed of two fully connected layers, to assess the prognostic risk for each patient.

After obtaining the prompt‐enhanced modality feature vectors $H_{i,(m)}^{W}$, $H_{i,(m)}^{R}$, and $H_{i,(m)}^{T}$, we designed three self‐attention modules to further explore the internal relationships of each modality feature and its association with prognosis. Each self‐attention module computes the correlation matrix within the features to dynamically adjust the weights of important features, as described by the following formula:

(7) $$\mathrm{Attention}\left(Q,K,V\right)=\mathrm{softmax}\left(\frac{QK^{T}}{\sqrt{d_{k}}}\right)V$$

where *Q*, *K*, *V* represent the query matrix, key matrix, and value matrix, respectively, while *d_k_* denotes the feature dimension used for normalization. Through the self‐attention mechanism, we can enhance the important features within the modality and mitigate the impact of redundant information.

After processing through the self‐attention modules, the feature vectors from each modality are concatenated into a fused feature *H_i_*, which is then input into the multi‐modal prognostic risk prediction network (Multi‐Modal SurvNet). This network consists of two fully connected layers, designed to further extract the nonlinear relationships of the fused features and predict each patient's risk of death and survival probability across different time intervals. The following provides a detailed description of the loss function used in FLARE.

Following the method of Chen et al. [9], we first divide the patients' overall survival time (measured in months on a continuous time scale) into four nonoverlapping intervals: [*t*_0_,*t*_1_),[*t*_1_,*t*_2_),[*t*_2_,*t*_3_),[*t*_3_,*t*_4_), where *t*_0_ =  0,  *t*_4_ =  ∞, and *t*_1_,*t*_2_,*t*_3_ define the quartiles of the event times for non‐censored patients. For each patient, their follow‐up time $T_{j,\;\mathrm{cont}\;}\in \left[0,\infty \right)$ is discretized into an event time interval index *T_j_*, as defined as below:

(8) $$T_{j}=r\;\mathrm{iff}\;T_{j,\;\mathrm{cont}\;}\in \left[t_{r},t_{r+1}\right)$$

To avoid confusion, the discretized true label of the j‐th patient is denoted as *Y_j_*. For patients with the fused feature vector *H_i_*, the risk function is modeled through the prediction layer, Multi‐Modal SurvNet, as follows:

(9) $$f_{\mathrm{hazard}\;}\;\left(r|H_{j}\right)=P\left(\;T_{j}=r|T_{j}\geq r,H_{j}\right)$$

The risk function is related to the survival function through the following relationship:

(10) $$\begin{array}{c} f_{\mathrm{surv}\;}\left(r|H_{j}\right)=P\left(T_{j}>r|H_{j}\right)\\ =\prod_{u=1}^{r}\left(1-f_{\mathrm{hazard}\;}\left(u|H_{j}\right)\right) \end{array}$$

To optimize the model parameters, we employed a log‐likelihood function based on a discrete survival model. Given the binary censoring status *c_j_*, the objective function is defined as:

(11) $$\begin{array}{c} L=-I=-c_{j}\cdot log\left(f_{\mathrm{surv}\;}\left(Y_{j}|H_{j}\right)\right)\\ \left.-\left(1-c_{j}\right)\cdot log\left(f_{\mathrm{surv}\;}\left(Y_{j}-1|H_{j}\right)\right)\right)\\ -\left(1-c_{j}\right)\cdot log\left(f_{\mathrm{hazard}\;}\left(Y_{j}|H_{j}\right)\right) \end{array}$$

In this framework, we assign *c_j_* =  1 to patients who survived beyond the follow‐up period, and *c_j_* =  0 to those who died at the time point *T*_*j*,*count*_. To further emphasize the contribution of uncensored patients during the training process, the final loss function is a weighted sum of two components:

(12) $$L_{\mathrm{surv}}=\left(1-\beta \right)\;\cdot L+\beta \cdot L_{\mathrm{uncersoned}}$$

The loss function for uncensored patients (the second component) is defined as:

(13) $$\begin{array}{c} \;L_{\mathrm{uncensored}\;}=-\left(1-c_{j}\right)\cdot log\left(f_{\mathrm{surv}\;}\left(Y_{j}-1|H_{j}\right)\right)\\ -\left(1-c_{j}\right)\cdot log\left(f_{\mathrm{hazard}\;}\left(Y_{j}|H_{j}\right)\right) \end{array}$$

## 5 Statistical Analysis

All statistical analyses were performed using R software (version 4.1.3) and SPSS (version 20). Continuous variables were compared between groups using the Wilcoxon rank‐sum test, while categorical variables were compared using Fisher's exact test or the chi‐squared test. Survival curves were generated using the Kaplan‐Meier method, with curve comparisons conducted using the log‐rank test in the R package ‘survminer’ (version 0.4.9). Cox regression analysis was performed for both univariate and multivariate analyses to estimate hazard ratios (HRs) and their corresponding 95% confidence intervals (CIs). The training and testing of deep learning models were conducted using Python (version 3.9.12) and PyTorch (version 2.1.0). Detailed model training parameters and hardware configurations can be found in the Supplementary Information.

## Conflicts of Interest

The authors declare no conflicts of interest.

## Supporting information




**Supporting File**: advs73818‐sup‐0001‐SuppMat.docx.

## Data Availability

The data for the FUSCC, FUZSH, and FUHSH cohorts were collected and organized through the internal databases of Fudan University Shanghai Cancer Center (FUSCC), Fudan University Zhongshan Hospital (FUZSH), and Fudan University Huashan Hospital (FUHSH), respectively. The publicly available TCGA‐COAD&READ cohort can be accessed and downloaded via the NIH Genomic Data Commons (GDC) Data Portal (https://portal.gdc.cancer.gov/). All internal datasets were authorized by the respective institutions and approved by their Ethics Review Boards. These datasets are restricted for use exclusively in this study and cannot be publicly shared. The training code for this study will be made available after the publication of the paper, exclusively for non‐commercial academic use. All deep learning methodologies and software details utilized in the research are meticulously documented, ensuring the paper remains accessible and comprehensible to a broader scientific audience.
