# Supplementary material for: Foundation Model‐Enabled Multimodal Deep Learning for Prognostic Prediction in Colorectal Cancer with Incomplete Modalities: A Multi‐Institutional Retrospective Study
Source: Adv Sci (Weinh). 2026 Jan 20;13(17):e10931. doi: 10.1002/advs.202510931 (PMC13042662; doi:10.1002/advs.202510931)
Supplement: Supplementary file 1 — Supporting File: advs73818‐sup‐0001‐SuppMat.docx. [file ADVS-13-e10931-s001.docx]

**Supplementary Information**

**1 More Heatmap Visualization**

|  | Original WSI | Heatmap |
| --- | --- | --- |
| Example 1 | 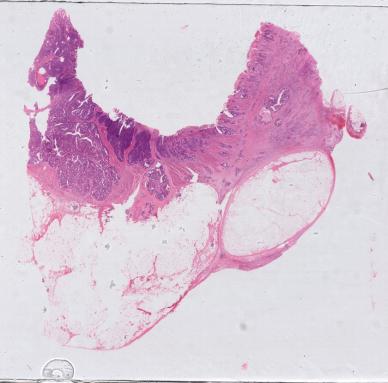 | 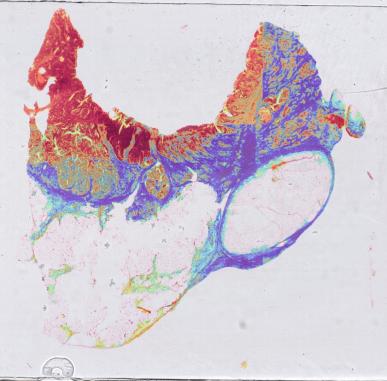 |
| Example 2 | 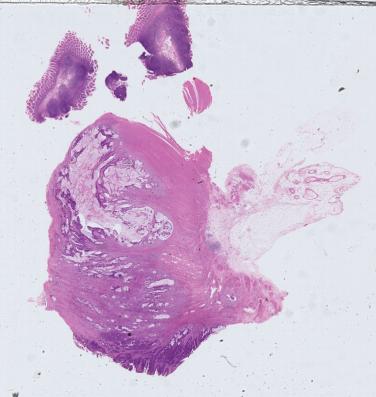 | 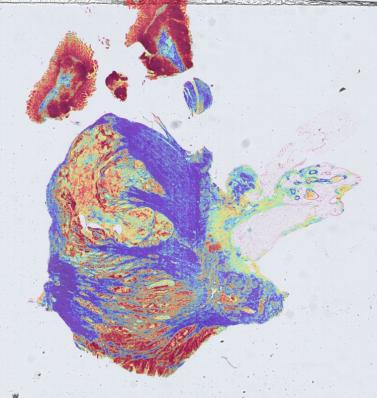 |
| Example 3 | 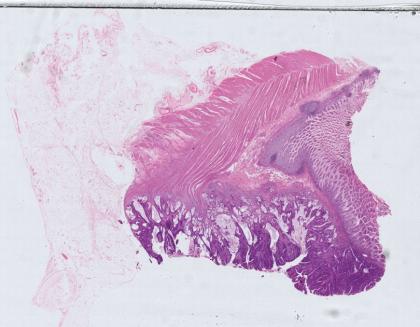 | 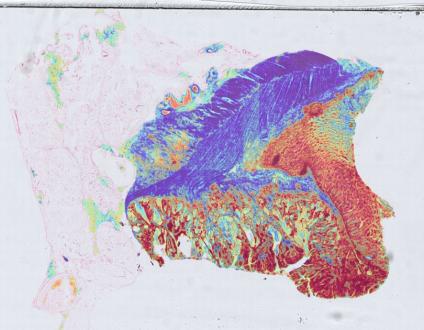 |
| Example 4 | 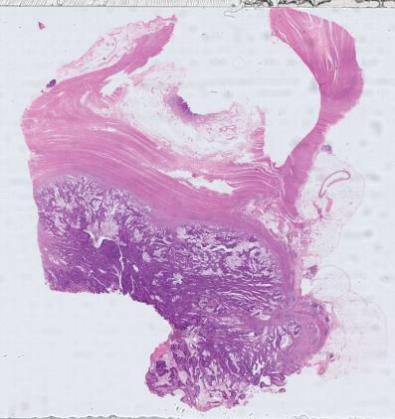 | 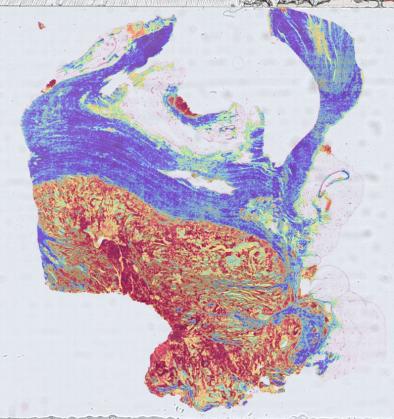 |
| Example 5 | 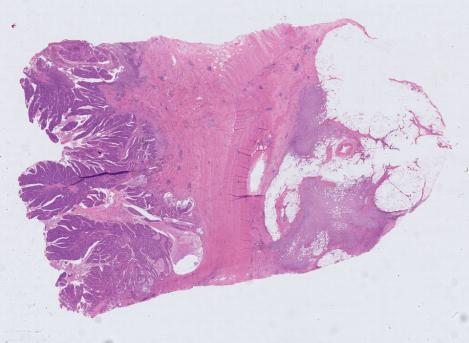 | 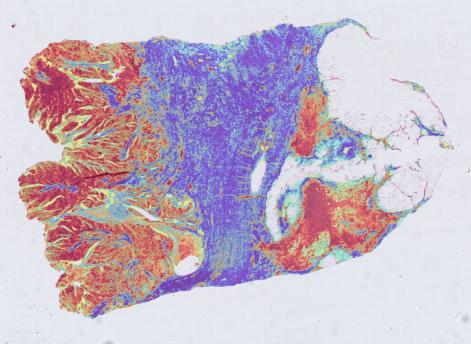 |
| Example 6 | 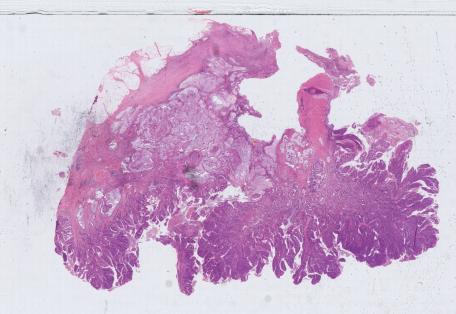 | 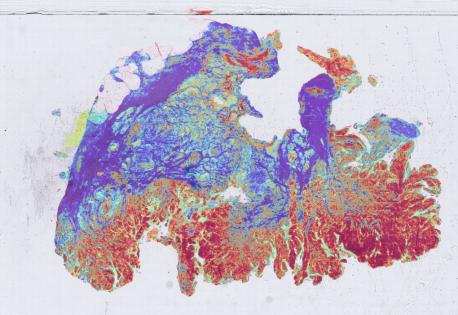 |
| Example 7 | 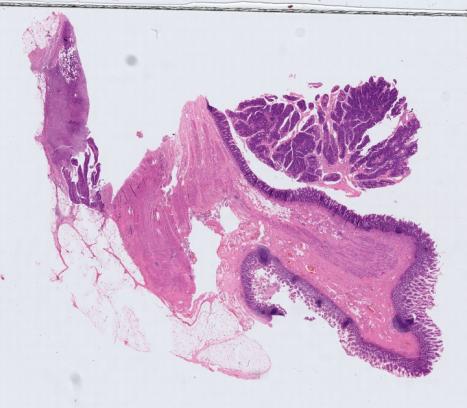 | 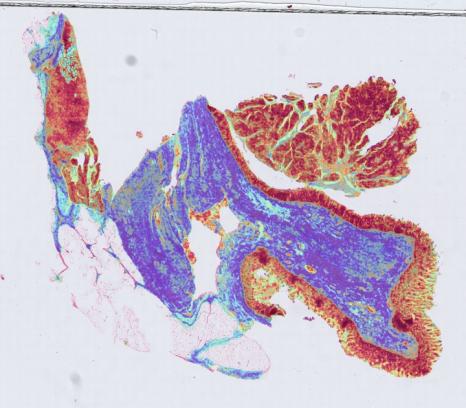 |

**Figure S1:** More heatmap visualization. The first column shows the histopathological WSI, and the second column displays the heatmap visualization based on attention scores of FLARE.

**2 Foundation Model for Feature Extraction**

**2.1 Introduction of PLIP**

PLIP [1] (Pathology Language–Image Pretraining) is a multimodal foundation model designed for pathology image analysis. The model is trained on the OpenPath dataset, which contains 208,414 pairs of pathology images and natural language descriptions. By fine-tuning the pre-trained CLIP [1] model through contrastive learning, the model is able to understand the relationship between images and text, enabling zero-shot classification, image retrieval, and other functionalities in various downstream applications, providing new tools and methods for pathology image analysis.

In this study, each WSI is first segmented into patches, and the image patches are processed through PLIP's visual encoder to obtain visual feature vectors, while the pathology reports are processed through PLIP's text encoder to obtain feature vectors, which are then used for subsequent feature aggregation and other operations.

**2.2 Introduction of MedSAM**

MedSAM [3] is a foundational model designed for medical image segmentation, aimed at performing general medical image segmentation tasks. It is trained on a large-scale dataset containing 1,570,263 pairs of images and masks, covering 10 imaging modalities and over 30 cancer types. By fine-tuning the SAM [4] and incorporating specific network architectures (including image encoder, prompt encoder, and mask decoder), it is capable of segmenting various anatomical structures and pathological regions based on user-provided prompts, such as bounding boxes.

In this study, the radiological images processed by MedSAM are used to obtain feature vectors for subsequent operations.

**2.3 Introduction of LinkBERT**

LinkBERT [5] is a pretraining method for language models that integrates cross-document knowledge by leveraging document links (such as hyperlinks and citation links). During pretraining, it treats the corpus as a document graph, creating input instances by placing linked documents within the same context, and is trained with two objectives: Masked Language Modeling (MLM) and Document Relationship Prediction (DRP). Experiments in both the general domain (using Wikipedia articles with hyperlinks for training) and the biomedical domain (using PubMed articles with citation links for training) show that LinkBERT performs exceptionally well on a variety of downstream tasks.

In this study, textual data such as pathology reports, radiology reports, clinical histories, and colonoscopy reports are processed by LinkBERT to obtain feature vectors for subsequent operations.

**3 Network Architecture and Training Details**

**3.1 Feature Aggregation Network Architecture**

Modal_Encoder_adapter:


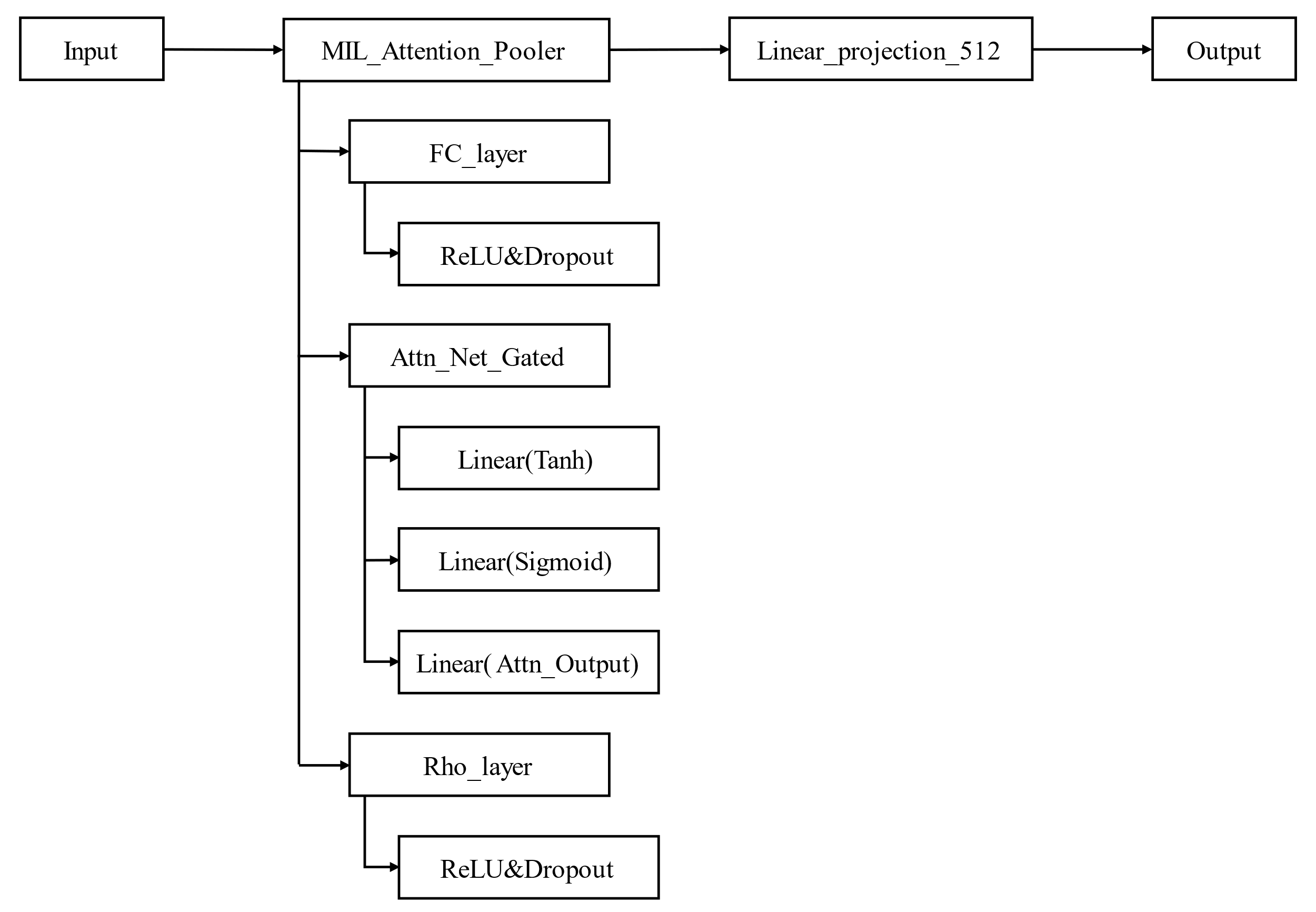


**Figure S2:** Feature aggregation network architecture. The module of feature aggregation is named Modal_Encoder_adapter, which consists of some basic linear layers, activation layers, etc.

**3.2 Modality Fusion Network Architecture**

Multimodal_Surv_Net_Encoder:


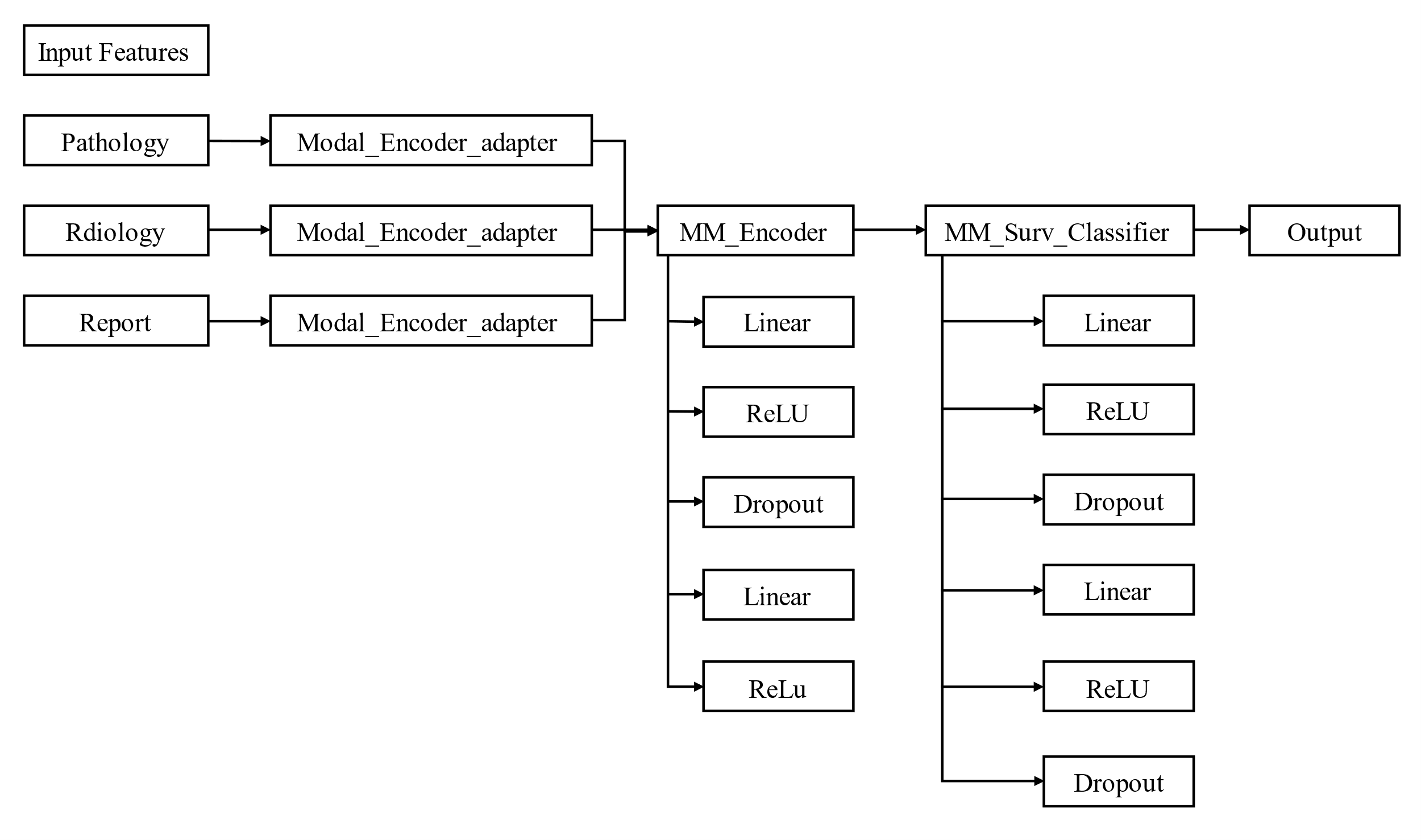


**Figure S3**: Modality fusion network architecture. "Pathology," "Radiology," and "Report" represent the features of each modality. "Modal_Encoder_adapter" is shown in Figure 2. "MM_Encoder" and "MM_Surv_Classifier" are composed of some basic linear layers and activation layers.

**3.3 Hyperparameter Settings**

**Table S1:** The hyperparameters used for model training.

| Hyperparameter | Data type | Setting | Description |
| --- | --- | --- | --- |
| --patch_input_dim | int | 512 | pathology feature input dimension |
| --rad_input_dim | int | 256 | radiology feature input dimension |
| --report_input_dim | int | 1024 | text report feature input dimension |
| --n_classes | int | 4 | Number of class |
| --seed | int | 42 | random seed |
| --k | int | 5 | number of cross-validation folds |
| --log_data | bool | True | TensorBoard |
| --opt | str | ‘adam’ | optimizer type |
| --batch_size | int | 1 | batch size |
| --gc | int | 32 | gradient accumulation steps |
| --max_epochs | int | 200 | maximum number of training epochs |
| --lr | float | 1e-4 | learning rate |
| --weight_con | float | 0.5 | weight coefficient |
| --bag_loss | str | ‘nll_surv’ | sliding level classification loss function |
| --label_frac | float | 1.0 | proportion of training labels |
| --bag_weight | float | 0.7 | weight coefficient for batch-level loss |
| --reg | float | 1e-4 | L2 regularization weight decay |
| --alpha_surv | float | 0.0 | weight coefficient for uncensored patients |
| --lambda_reg | float | 1e-4 | L1 regularization strength |

All training was conducted on an NVIDIA GeForce RTX 4090 GPU with 24GB of memory.

**4 Supplementary Results**

**4.1 Comparative Experiments——OS**

**Tabel S2:** FLARE significantly outperforms clinical model and other comparative models in C-index results for OS prediction tasks across four cohorts.

|  | Data Split | *Internal Validation* | *External Validation* | *External Validation* | *External Validation* |
| --- | --- | --- | --- | --- | --- |
|  | Hospital | FUSCC | FUZSH | FUHSH | TCGA-COAD/READ |
|  | Task | OS | OS | OS | OS |
| Clinical Model | Clinical Cox [11] | 0.713±0.0058 | 0.618±0.0057 | 0.544±0.0051 | 0.674±0.0065 |
| Single-Modality Model | Pathology_only [12] | 0.748±0.0035 | 0.672±0.0063 | 0.678±0.0053 | 0.584±0.0042 |
|  | Radiology_only [12] | 0.735±0.0056 | 0.650±0.0061 | 0.647±0.0070 | - |
|  | Report_only [12] | 0.751±0.0051 | 0.683±0.0055 | 0.664±0.0054 | 0.553±0.0062 |
| Multi-Modality Model | Generating-based [13] | 0.733±0.0068 | 0.621±0.0057 | 0.615±0.0063 | 0.612±0.0037 |
|  | Feature-aggregation-Average [14] | 0.749±0.0056 | 0.685±0.0054 | 0.668±0.0063 | 0.672±0.0067 |
|  | Feature-aggregation-Attention [15] | 0.764±0.0045 | 0.723±0.0058 | 0.708±0.0044 | 0.658±0.0058 |
|  | **Ours** | **0.812±0.0060** | **0.790±0.0057** | **0.742±0.0054** | **0.730±0.0067** |

**4.2 Comparative Experiments——PFS**

**Table S3:** FLARE significantly outperforms clinical model and other comparative models in C-index results for PFS prediction tasks across four cohorts.

|  | Data Split | *Internal Validation* | *External Validation* | *External Validation* | *External Validation* |
| --- | --- | --- | --- | --- | --- |
|  | Hospital | FUSCC | FUZSH | FUHSH | TCGA-COAD/READ |
|  | Task | PFS | PFS | PFS | PFS |
| Clinical Model | Clinical Cox [11] | 0.701±0.0050 | 0.658±0.0049 | 0.672±0.0065 | 0.651±0.0047 |
| Single-Modality Model | Pathology_only [12] | 0.789±0.0056 | 0.728±0.0075 | 0.733±0.0050 | 0.593±0.0071 |
|  | Radiology_only [12] | 0.774±0.0064 | 0.683±0.0039 | 0.696±0.0038 | - |
|  | Report_only [12] | 0.786±0.0063 | 0.725±0.0057 | 0.741±0.0070 | 0.567±0.0043 |
| Multi-Modality Model | Generating-based [13] | 0.77±0.0053 | 0.684±0.0054 | 0.687±0.0067 | 0.624±0.0068 |
|  | Feature-aggregation-Average [14] | 0.795±0.0052 | 0.713±0.0047 | 0.753±0.0040 | 0.654±0.0076 |
|  | Feature-aggregation-Attention [14] | 0.838±0.0054 | 0.765±0.0039 | 0.781±0.0045 | 0.663±0.0060 |
|  | **Ours** | **0.901±0.0049** | **0.801±0.0064** | **0.829±0.0062** | **0.725±0.0045** |

**4.3 Comparison Model Development and Implementation Details**

**4.3.1 Clinical Cox Baseline Model**

The clinical baseline model was developed using Cox regression [11]. It incorporated AJCC/TNM staging and other crucial clinical factors (e.g., age), which were selected via a univariate Cox regression analysis performed on the FUSCC training cohort, as illustrated in Figure 2d. This model was trained on the FUSCC cohort and subsequently validated on external cohorts to assess its generalizability.

**4.3.2 Single-Modal Baseline Models**

For the single-modal baseline models, we employed three distinct models, each trained on an individual modality for prognosis prediction. The network architecture was adapted from CLAM [12]. As these are unimodal models, the feature diversity loss was modified to its original clustering loss formulation to suit the single-modality learning context.

**4.3.3 Multimodal Baseline Models**

We included three mainstream multimodal approaches for comparison:

**Generative Models**:​ These models first generate missing modalities from the available ones before performing fusion. The specific generating-based model implemented the fusion method described in [13].

**Feature Averaging Models**:​ This approach represents a simple fusion strategy, implemented by averaging the features extracted from each modality. This feature-aggregation-average method follows the technique outlined in [14].

**Attention Aggregation Models**:​ This more sophisticated method performs a weighted fusion of modality features using an attention mechanism. Our implementation of this feature-aggregation-attention approach was based on the methodology presented in [15].

**5 Bioinformatics Processing Tools**

**5.1 CIBERSORT**

CIBERSORT [6,7] is a method used to characterize cellular composition from tissue expression profiles, accurately inferring the relative proportions of various cell subsets in complex tissues. It is particularly advantageous in analyzing mixtures containing noise, unknown components, and closely related cell types. CIBERSORT estimates cell type proportions by referencing a gene expression feature matrix. Its deconvolution model is based on linear equations, representing the mRNA mixture as a weighted sum of cell type-specific gene expression profiles (GEPs). It uses linear support vector regression (SVR), which adapts to select genes, is robust to noise, and handles multicollinearity through a penalty function. The method uses the LM22 leukocyte gene signature matrix, which contains 547 genes and can differentiate 22 human hematopoietic cell phenotypes. It is constructed through differential expression analysis and enables more robust cell type identification.

Regarding the use of CIBERSORT, the first step is to obtain gene expression data, which can come from high-throughput sequencing (e.g., RNA-seq) or gene microarray technologies, ensuring data quality and integrity. Next, sample information should be determined for accurate subsequent analysis and interpretation. Finally, the data is input into the CIBERSORT official software platform for processing.

**5.2 Gene set enrichment analysis (GSEA) was performed on the KEGG biological database and GO database for gene pathway enrichment analysis.**

GSEA [8] (Gene Set Enrichment Analysis) is a powerful analytical method for interpreting gene expression data. It overcomes many of the limitations of traditional single-gene analyses by focusing on gene sets (groups of genes that share common biological function, chromosomal location, or regulation), revealing the biological significance behind gene expression data.

KEGG [9] (Kyoto Encyclopedia of Genes and Genomes) is a knowledge base that links genomic information with higher-level functional data. By integrating cellular process knowledge and standardized gene annotations, it provides systematic analysis of gene functions. KEGG offers various tools to support research and is widely used in biomedical fields to assist researchers in understanding gene functions, pathway mechanisms, disease causes, and more.

The GO [10] (Gene Ontology) database, constructed by the Gene Ontology Consortium, contains three independent ontologies: biological processes, molecular functions, and cellular components. Its purpose is to address interoperability issues in genomic databases caused by differences in gene and protein naming systems, and to facilitate the integration and sharing of biological knowledge through the establishment of a common language. The database is continually evolving to meet the rapidly changing demands of biological knowledge.

The GSEA method primarily focuses on gene sets (groups of genes that share common biological functions, chromosomal locations, or regulations) and aims to determine the enrichment of these gene sets in sample classification. Its core principle is to calculate the Enrichment Score (ES) to assess the distribution of a gene set within the entire ranked gene list. The ES reflects the degree of overrepresentation of the gene set at the two extremes of the ranked list (high or low expression). Genes are ranked based on the correlation of their expression differences between two sample classes, forming a ranked list L. For a predefined gene set S (e.g., from the KEGG or GO databases), GSEA calculates its ES to determine whether this gene set is associated with phenotypic differences in the samples. If gene set members tend to cluster at one end of the list, the ES will be high; conversely, for a randomly distributed gene set, the ES will be low.

**6 Further Ablation Study of Branch and Feature Visualization**

We performed ablation experiments regarding the choice of branch number. On the FUSCC OS internal validation set, we compare single, dual, triple, and four branch attention architectures, as reported in the **Table S4**. The single branch model achieves the lowest performance, with a C-index of 0.798. Increasing to a dual branch setting improves the C-index to 0.807. Extending to a triple branch architecture yields a further gain, with a C-index of 0.812. However, when we increase the number of branches to four, performance declines and the C-index drops to 0.804. These results show that increasing architectural complexity within a moderate range can improve performance, but that excessive complexity leads to saturation and even degradation. In other words, performance does not increase monotonically with the number of branches, and three branches provide a good balance between model capacity and overfitting for this task.

To qualitatively assess feature diversity, we visualized the high dimensional features from all three branches using UMAP for each modality. As shown in the **Figure S4**, the projected features form three well separated clusters. Each cluster is predominantly composed of features from one specific branch, with minimal overlap between clusters. This pattern indicates that the three branches do not simply learn redundant representations. Instead, each branch specializes in a different aspect of the data. The fact that these feature spaces are largely distinct supports our design that the final prediction is based on the fusion of three complementary information streams.

**Table S4** Ablations of attention branches.

| Method | FUSCC OS |
| --- | --- |
| Single branch | 0.798±0.0082 |
| Double branch | 0.807±0.0076 |
| Three branched | 0.812±0.0060 |
| Four branches | 0.804±0.0065 |

| UMAP of pathology branch | UMAP of radiology branch | UMAP of report branch |
| --- | --- | --- |
| 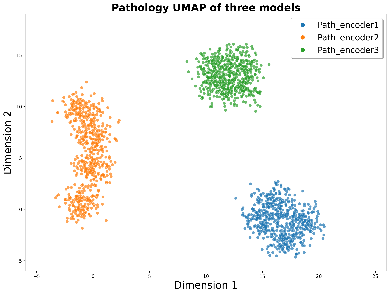 | 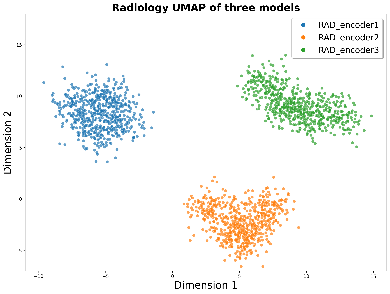 | 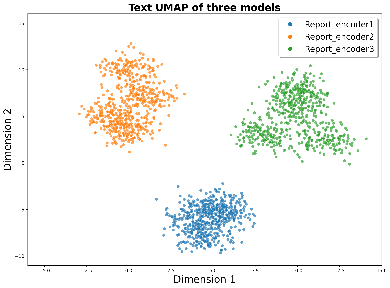 |

**Figure S4**: UMAP visualization of the high-dimensional features extracted by the three branches of our proposed model.

**7 Patient Characteristics of Our Study**

| **Table S5.** Patient characteristics of our study cohorts | | | | | | |
| --- | --- | --- | --- | --- | --- | --- |
| **Name** | **Levels** | **FUSCC (N=839)** | **FUZSH (N=154)** | **FUHSH (N=87)** | | **TCGA (N=584)** |
| **Age** | Mean±SD | 61.6 ± 11.9 | 63.5 ± 10.4 | | 58.5 ± 10.5 | 65.8 ± 12.3 |
| **Sex** | Female | 328 (39.1%) | 62 (36.7%) | | 45 (51.7%) | 266 (45.5%) |
|  | Male | 511 (60.9%) | 107 (63.3%) | | 42 (48.3%) | 318 (54.4%) |
| **pTstage** | T1 | 2 (0.2%) | 12 (7.1%) | | 1 (1.1%) | 20 (3.4%) |
|  | T2 | 21 (2.5%) | 30 (17.8%) | | 14 (16.1%) | 107 (18.3%) |
|  | T3 | 725 (86.4%) | 112 (66.3%) | | 58 (66.7%) | 394 (67.5%) |
|  | T4 | 91 (10.8%) | 15 (8.9%) | | 14 (16.1%) | 61 (10.4%) |
|  | Tis | 0 (0%) | 0 (0%) | | 0 (0%) | 2 (0.3%) |
| **Nstage** | N0 | 412 (49.4%) | 97 (57.4%) | | 42 (48.3%) | 334 (57.2%) |
|  | N1 | 293 (34.9%) | 59 (34.9%) | | 37 (42.5%) | 414 (24.1%) |
|  | N2 | 134 (16%) | 13 (7.7%) | | 8 (9.2%) | 107 (18.3%) |
|  | NX | 0 (0%) | 0 (0%) | | 0 (0%) | 2 (0.3%) |
| **Mstage** | M0 | 839 (100%) | 166 (98.2%) | | 86 (98.9%) | 444 (78%) |
|  | M1 | 0 (0%) | 3 (1.8%) | | 1 (1.1%) | 82 (14%) |
|  | MX | 0 (0%) | 0 (0%) | | 0 (0%) | 58 (9.9%) |
| **AJCC** | Stage I | 1 (0.1%) | 35 (20.7%) | | 36 (41.9%) | 198 (34.5%) |
|  | Stage Ⅱ | 390 (46.5%) | 60 (39.0%) | | 43 (50%) | 155 (27.0%) |
|  | Stage Ⅲ | 37 (4.4%) | 27 (17.5%) | | 1 (50.6%) | 72 (12.5%) |
|  | Stage Ⅳ | 0 (0%) | 3 (1.8%) | | 1 (1.1%) | 83 (14.2%) |
|  | Unknown | 0 (0%) | 0 (0%) | | 0 (0%) | 19 (3.3%) |
| **Vital status** | Alive | 712 (84.9%) | 161 (95.3%) | | 80 (92%) | 466 (79.8%) |
|  | Dead | 127 (15.1%) | 8 (4.7%) | | 7 (8%) | 118 (20.2%) |
| **Recurrence** | No | 650 (77.5%) | 138 (81.7%) | | 72 (82.8%) | 352 (60.3%) |
|  | Yes | 189 (22.5) | 15 (8.9%) | | 15 (17.2%) | 71 (12.2%) |
|  | Unknown | 0 (0%) | 16 (9.5%) | | 0 (0%) | 161 (27.6) |

**Reference**

[1]. Huang, Z. *et al.* A visual-language foundation model for pathology image analysis using medical Twitter. *Nat Med* **29**, 2307–2316 (2023).

[2]. Radford, A. *et al.* Learning transferable visual models from natural language supervision. In *Proceedings of the 38th International Conference on Machine Learning (ICML 2021)*, **PMLR** 139, 8748–8763 (2021).

[3]. Ma, J. *et al.* Segment anything in medical images. *Nat Commun* **15**, 654 (2024).

[4]. Kirillov, A. *et al.* Segment Anything. In *Proceedings of the IEEE/CVF International Conference on Computer Vision (ICCV 2023)*, 4015–4026 (2023).

[5]. Yasunaga, M., Leskovec, J. & Liang, P. LinkBERT: Pretraining language models with document links. In *Proceedings of the 60th Annual Meeting of the Association for Computational Linguistics (Volume 1: Long Papers)*, 8003–8016 (Association for Computational Linguistics, 2022).

[6]. Newman, A. M. *et al.* Robust enumeration of cell subsets from tissue expression profiles. *Nat Methods* **12**, 453–457 (2015).

[7]. Newman, A. M. *et al.* Determining cell type abundance and expression from bulk tissues with digital cytometry. *Nat Biotechnol* **37**, 773–782 (2019).

[8]. Subramanian, A. *et al.* Gene set enrichment analysis: a knowledge-based approach for interpreting genome-wide expression profiles. *Proc Natl Acad Sci USA* **102**, 15545–15550 (2005).

[9]. Kanehisa, M. & Goto, S. KEGG: Kyoto encyclopedia of genes and genomes. *Nucleic Acids Res* **28**, 27–30 (2000).

[10]. Ashburner, M. *et al.* Gene ontology: tool for the unification of biology. *Nat Genet* **25**, 25–29 (2000).

[11]. Cox, D. R. Regression models and life-tables. *Journal of the Royal Statistical Society: Series B (Methodological)* **34**, 187–202 (1972).

[12]. Lu, M. Y. *et al.* Data-efficient and weakly supervised computational pathology on whole-slide images. *Nat Biomed Eng* **5**, 555–570 (2021).

[13]. Qian, X. *et al.* A multimodal machine learning model for the stratification of breast cancer risk. *Nat Biomed Eng* **9**, 356–370 (2025). doi:10.1038/s41551-024-01302-7.

[14]. Qian, X. *et al.* Prospective assessment of breast cancer risk from multimodal multiview ultrasound images via clinically applicable deep learning. *Nat Biomed Eng* **5**, 522–532 (2021).

[15]. Volinsky-Fremond, S. *et al.* Prediction of recurrence risk in endometrial cancer with multimodal deep learning. *Nat Med* **30**, 1962–1973 (2024).
